# Clinical and genetic risk factors underlying severe consequence identified in 75 families with unilateral high myopia

**DOI:** 10.1186/s12967-024-04886-5

**Published:** 2024-01-19

**Authors:** Yi Jiang, Xueshan Xiao, Wenmin Sun, Yingwei Wang, Shiqiang Li, Xiaoyun Jia, Panfeng Wang, J. Fielding Hejtmancik, Qingjiong Zhang

**Affiliations:** 1 State Key Laboratory of Ophthalmology, Zhongshan Ophthalmic Center, Sun Yat-Sen University, Guangdong Provincial Key Laboratory of Ophthalmology and Visual Science, 54 Xianlie Road, Guangzhou, 510060 China; 2 Ophthalmic Molecular Genetics Section, Ophthalmic Genetics and Visual Function Branch, National Eye Institute, Rockville, MD 20852 USA

**Keywords:** Unilateral high myopia, Genetic, Peripheral retinal examination, Anisometropia, High myopia

## Abstract

**Backgrounds:**

Unilateral high myopia (uHM), commonly observed in patients with retinal diseases or only with high myopia, is frequently associated with amblyopia with poor prognosis. This study aims to reveal the clinical and genetic spectrum of uHM in a large Chinese cohort.

**Methods:**

A total of 75 probands with simplex uHM were included in our Pediatric and Genetic Eye Clinic. Patients with significant posterior anomalies other than myopic fundus changes were excluded. Variants were detected by exome sequencing and then analyzed through multiple-step bioinformatic and co-segregation analysis and finally confirmed by Sanger sequencing. Genetic findings were correlated with associated clinical data for analysis.

**Results:**

Among the 75 probands with a mean age of 6.21 ± 4.70 years at the presentation, myopic fundus of C1 and C2 was observed in 73 (97.3%) probands. Surprisingly, specific peripheral changes were identified in 63 eyes involving 36 (48.0%) probands after extensive examination, including peripheral retinal avascular zone (74.6%, 47/63 eyes), neovascularization (54.0%), fluorescein leakage (31.7%), peripheral pigmentary changes (31.7%), and others. Exome sequencing identified 21 potential pathogenic variants of 13 genes in 20 of 75 (26.7%) probands, including genes for Stickler syndrome (*COL11A1* and *COL2A1*; 6/20), FEVR (*FZD4*, *LRP5*, and *TSPAN12*; 5/20), and others (*FBN1, GPR179, ZEB2*, *PAX6, GPR143*, *OPN1LW, FRMD7,* and *CACNA1F*; 9/20). For the peripheral retinal changes in the 20 probands, variants in Stickler syndrome-related genes were predominantly associated with retinal pigmentary changes, lattice degeneration, and retinal avascular region, while variants in genes related to FEVR were mainly associated with the avascular zone, neovascularization, and fluorescein leakage.

**Conclusions:**

Genetic defects were identified in about one-fourth of simplex uHM patients in which significant consequences may be hidden under a classic myopic fundus in up to half. To our knowledge, this is the first systematic genetic study on simplex uHM to date. In addition to routine care of strabismus and amblyopia, careful examination of the peripheral retina and genetic screening is warranted for patients with uHM in order to identify signs of risk for retinal detachment and other complications and provide meaningful genetic counseling.

**Supplementary Information:**

The online version contains supplementary material available at 10.1186/s12967-024-04886-5.

## Background

Anisometropia is a condition characterized by imbalanced ocular development of the two eyes, despite both eyes being exposed to similar genetic and environmental influences. Unilateral high myopia (uHM), a specific form of anisometropia, refers to patients with cycloplegic spherical equivalents of at least − 5.00 to − 6.00D in the highly myopic eye and a difference of 5.00 diopters or more between the two eyes [1], although variable criteria have also been used [2]. Unilateral high myopia is easily missed by their parents because of the relatively normal contralateral eye, although it is generally early-onset and frequently associated with serious consequences such as amblyopia with poor treatment outcomes, strabismus, or retinal detachment [2–4]. It may be classified into complex and simplex forms depending on whether it is associated with other obvious ocular or systemic diseases. Complex uHM is known to be associated with corneal opacity, congenital lens abnormalities, uveal coloboma, morning glory syndrome, Straatsma syndrome, Stickler syndrome, familial exudative vitreoretinopathy, retinopathy of prematurity, and other complications that are usually easily recognizable [2, 5–10]. On the other hand, simplex uHM refers to uHM without noticeable ocular or systemic changes except for myopic posterior fundus on routine examination. Clinical analysis of simplex uHM has been rare [1, 4, 11]. Simplex uHM is often missed and rarely described in children not only because of insignificant myopic posterior fundus but also due to the compensatory visual acuity provided by the better eye. However, the disastrous diseases may hide in presumed simplex uHM, in addition to amblyopia or strabismus. Therefore, the systematic evaluation of simplex uHM may contribute to the early detection of risk signs and further improvements in clinical management.

Genetic factors have been shown to play a major role in early-onset high myopia (eoHM), [12] in which variants in RetNet genes could be detected in about 24% of eoHM families [13, 14] while those in eoHM genes contributed to about 9% of eoHM families [15–17] based on our previous studies on bilateral eoHM. Although genetic factors have also been suggested for uHM [2, 11, 18–20], the exact genetic defects rarely have been reported for uHM, especially for simplex uHM. Recently, high myopia in one eye has been found in 11% of obligate female carriers with unique haplotypes in *OPN1LW* that are a common cause of bilateral eoHM in males [16], suggesting that systematic analysis of other genes associated with ocular diseases may uncover additional genetic defects associated with uHM.

In the current study, a total of 75 unrelated probands with simplex uHM were recruited from the pediatric and genetic eye clinic at the Zhongshan Ophthalmic Center. Systematic ocular examinations were performed to reveal subtle clinical risk signs and exome sequencing of genomic DNA was performed to detect genetic variants. Potential pathogenic variants in 13 genes were identified in 26.7% (20/75) of probands, with majority being related to FEVR or Stickler syndrome. Notably, the molecular diagnostic rate of uHM is comparable to that of bilateral eoHM, indicating a significant role of monogenetic factors in the development of uHM. Those probands, with typical C1 (77.3%) and C2 (20.0%) myopic fundus without noticeable additional signs, were presumed to have simplex uHM at the initial routine clinical examination. Surprisingly, extensive additional examination including ultra-wide-field imaging and fundus fluorescein angiography (FFA) imaging revealed peripheral fundus changes in 48% of these cases, including color changes (pigmentary changes, hypopigmentation, degeneration), vascular abnormalities (avascular zone, neovascularization, fluorescein leakage), and fibrous proliferation. Our data highlight the importance of performing comprehensive peripheral retinal examinations and clinical genetic testing in patients with simplex or complex uHM in order to promptly detect the potential risk factors, such as retinal detachment and provide genetic counseling when needed, in addition to care of the strabismus and amblyopia.

## Methods

### Patient enrollment

This study was approved by the institutional review board of Zhongshan Ophthalmic Center. All patients with uHM and their family members in the study were enrolled from the Pediatric and Genetic Eye Clinic, Zhongshan Ophthalmic Center, Guangzhou, and evaluated by a senior ophthalmologist (Zhang Q). Based on the initial complaint and routine ophthalmic examination the preliminary diagnosis of probands from 75 unrelated families was simplex uHM that was defined as follows [1, 2]: (1) the highly myopic eye had a cycloplegic refraction (spherical equivalent) ≤ − 6.00 diopters (D) (or ≤ − 5.00 D when the age of a proband is less than 7 years old) while the fellow eye did not have such refraction; (2) The absolute difference of refraction between highly myopic eye and the fellow eye should be at least 5 diopters (≥ 5 D); (3) Exclusion of diseases involved in cornea, iris, and lens; (4) Exclusion of retinal diseases except for myopic fundus changes based on major complaint reported by the patient or their legal guardians and routine fundus examination; (5) Individuals with eoHM in both eyes were excluded. Prior to collecting peripheral venous blood samples and clinical data, all participants or their legal guardians underwent informed consent in accordance with the principles of the Declaration of Helsinki. The genomic DNA was prepared from the peripheral venous blood as previously described [13].

### Variants identification and evaluation

Whole-exome sequencing (WES), or targeted exome-sequencing (TES) on 736 eye-panel genes was conducted on the genomic DNA from the 75 probands with uHM, using methods described in our previous study [17]. For WES, the Agilent SureSelect Human All Exon Enrichment Kit (Agilent, Santa Clara, CA, United States) was used to construct the whole-exome library. The library quality assessment was performed using Agilent Technologies 2100 Bioanalyzer and library sequencing was conducted using Illumina HiSeq platform (Illumina, San Diego, CA, United States) with an average depth of at least 125-fold. The clean reads were mapped to the human reference genome hg19 with Burrows-Wheeler Aligner (BWA) software (http://bio-bwa.sourceforge.net/). Subsequently, variant calling was performed using GATK (https://gatk.broadinstitute.org/hc.en-us) and SAMTOOLS (http://samtools.sourceforge.net/) implementing Bayesian approaches, and annotation of the detected variants was performed using ANNOVAR (http://annovar.openbioinformatics.org/en/latest/).

For TES, through the Bioruptor Plus (Diagenode, Liege, Belgium), genomic DNA Fragments of approximately 200 base-pair (bp) were obtained. Subsequently, the fragments were subject to the KAPA HTP Library Preparation Kit (Roche, Basel, Switzerland) to generate a paired-end library. For the library capture, the NimbleGen SeqCap EZ Choice Library SR V5 kit (Roche) was utilized, and then the libraries were sequenced using NextSeq550 Mild output v2 kit (150 bp paired-end) on an Illumina Nextseq550 Analyzer (Illumina, San Diego, CA). Data analysis, including variant calling, annotation, and screening was conducted utilizing Strand NGS software (Karnataka, India). The genes included in the target gene panels are listed in Additional file 1: Table S1 as described in our previous study [17].

The detected variants were filtered through multiple steps of bioinformatic analysis as previously described. Firstly, variants with low sequencing quality (coverage depth less than 5) or with minor allele frequency (MAF) of more than 1% in the gnomAD database (https://gnomad.broadinstitute.org/) were excluded. Subsequently, the potential effect of missense variants was evaluated using five in silico prediction tools, including SIFT (http://sift.jcvi.org/), Polyphen-2 (http://genetics.bwh.harvard.edu/pph2/), PROVEAN (http://provean.jcvi.org/seq_submit.php), CADD (http://cadd.gs.washington.edu), and REVEL (https://sites.google.com/site/revelgenomics/). Then, the influence of variants on splicing signals was computationally examined by an online splicing prediction tool: Human splicing finder program (HSF, https://hsf.genomnis.com/mutation/analysis). Additionally, these variants were evaluated by a comparison analysis of the gnomAD database and the Human Gene Mutation Database (HGMD). The variants were evaluated according to the American College of Medical Genetics and Genomics (ACMG) and the Association for Molecular Pathology (AMP) criteria. Then, the residual variants, including pathogenic, likely pathogenic, and variants of uncertain significance, were confirmed by Sanger sequencing as described previously [21], for which the primers were designed using primer 3 (http://primer3.ut.ee/). Finally, co-segregation analysis was performed on available family members.

### Clinical data collection

General clinical information of all participants including age, sex, and first symptom was collected. Participants included in this study underwent routine ophthalmological examinations and necessary additional specific tests, including fundus photography, RetCam digital photography, scanning-laser ophthalmology (SLO), optical coherence tomography (OCT), fundus fluorescein angiography (FFA), and the electroretinogram (ERG). Based on fundus photography, SLO, Retcam, FFA, and OCT results, the peripheral retinal abnormalities were evaluated by two ophthalmologists. The evaluation on peripheral retina mainly considered color changes (pigmentary changes, hypopigmentation, degeneration), and vascular morphology (avascular zone, neovascularization, vascular leakage), and fibrous proliferation, as described in previous studies [22–25]. The comprehensive list of peripheral retinal changes observed in this study and represent image of each peripheral retinal changes could be found in Additional file 3: Fig. S3 and Table S6. In addition, grading of high myopia was based on the classification proposed by Ohno-Matsui et al. [26], where the myopic fundi were classified into five categories: Category 0 (no myopic retinal degenerative lesion), Category 1 (tessellated fundus), Category 2 (chorioretinal atrophy), Category 3 (patchy chorioretinal atrophy), Category 4 (macular atrophy). Foveal hypoplasia grading was performed according to the Leicester Grading System [27]. Patients with FEVR were staged based on the FEVR classification scheme proposed by Pendergast and Trese [28].

### Follow-up observation on progress of visual acuity after intervention

Follow-up observation on progress of visual acuity was through medical record review, telephone interviews, and outpatient follow-up visits. Of the 75 uHM probands, 38 had follow-up clinical data on wear of glasses together with patching therapy. The baseline date was defined as the date of the proband’s first treatment and the last follow-up date was defined as the date of the last measurement of best corrected visual acuity (BCVA). Each participant was advised to continuously wear spectacles for optical correction, with replacement of glasses in follow-up visit, if necessary. Additionally, the relative healthy eye was recommended to be patched for at least 4 h each day for at least five days a week, while in the meantime the highly myopic eye was encouraged to perform amblyopia training exercises once the healthy eye was patched. The patients were also advised to have regular follow up visit every six months. The mean follow-up time was 24.55 ± 20.17 months.

### Statistical analysis

All statistical analyses were performed using IBM SPSS statistics software v26.0 (IBM Corporation, Armonk, NY, USA). Clinical data were compared between the highly myopic eye and the fellow eye using a *Wilcoxon signed-rank test* or *Fihser’s exact test.* The comparison of the BCVA within the same uHM patient group at different time points was evaluated using the *Wilcoxon signed-rank test* or *paired Student’s t-test*. The refractive difference between the highly myopic eye and the fellow eye with the corresponding difference in total axial length was compared by linear regression. A *P value* less than 0.05 is taken to indicate statistical significance.

## Results

### The clinical characteristics of uHM

**Fig. 1:**
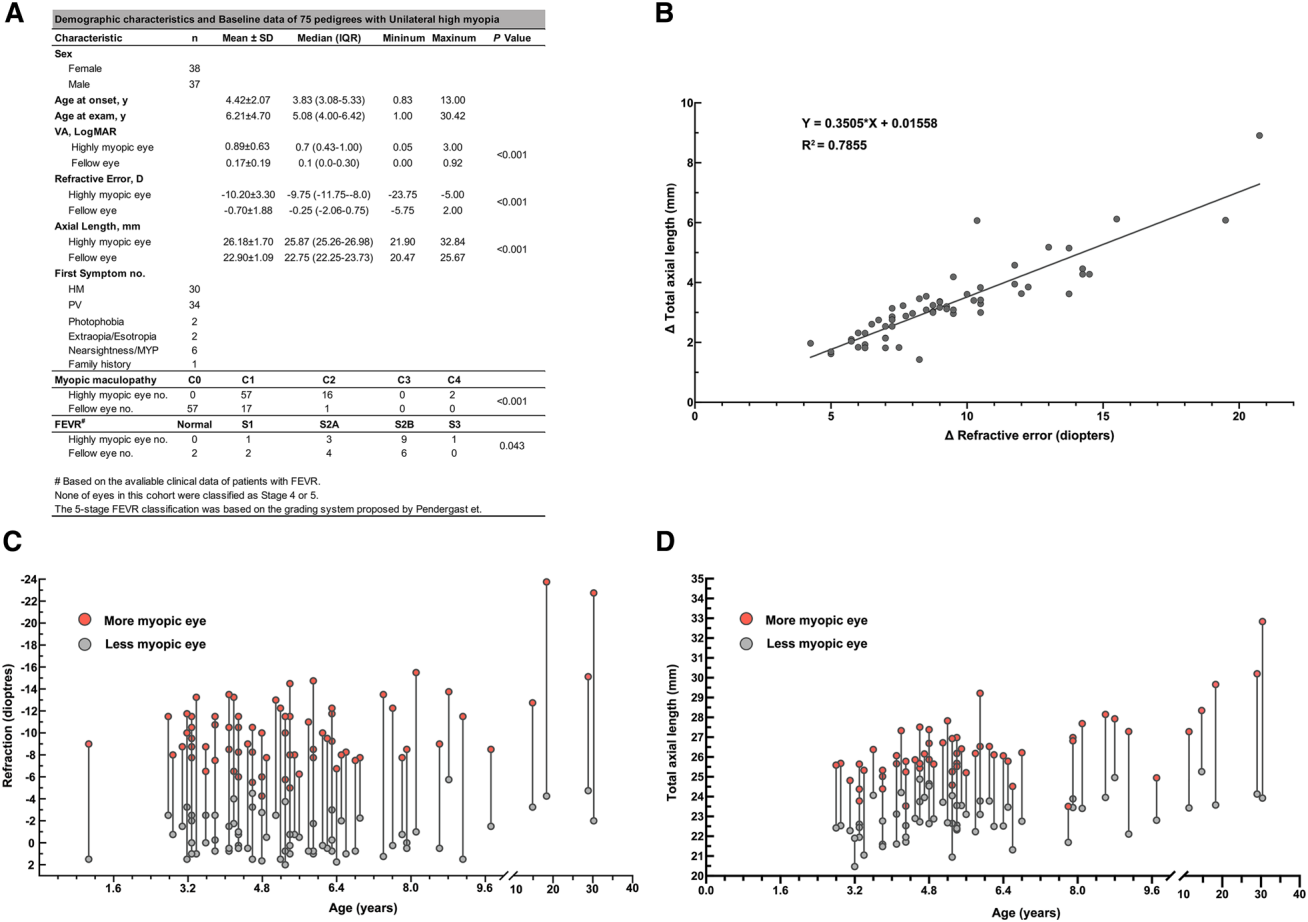
The demographic characteristics and clinical data of 75 pedigrees with unilateral high myopia in this study. **A** Demographic data and clinical characteristics including sex, age, visual acuity, refraction error, axial length, first symptom, myopic maculopathy classification, and FEVR stage classification. **B** Correlation of refraction error difference (diopters) and axial length difference (millimeters) between the highly myopic and contralateral eyes (R^2^ = 0.79, *P* < 0.0001). **C** Distribution of available refraction data in diopters of patients with unilateral high myopia in this study. **D** Distribution of available axial length in millimeters of patients with unilateral high myopia in this study. The red circles represent the highly myopic eyes, and the gray circles represent the fellow eye

The 75 unrelated probands initially diagnosed as uHM were recruited in the current study. The general clinical findings of these 75 uHM patients were presented in Fig. 1 and Additional file 1: Table S2. Of the 75 probands, 38 were female (50.7%) and 37 were male (49.3%), with a mean age at onset of 4.42 ± 2.07 years (median 3.83, interquartile range (IQR) 3.08 to 5.33 years) (Fig. 1A and Additional file 1: Table S2). At the first examination, the majority of the probands (71/75) were children except for four who were more than 12 years of age. The mean age at examination was 6.21 ± 4.70 years (median 5.08, IQR 4.00 to 6.42) (Fig. 1A). The most common initial symptoms were HM (40%, 30/75) and impaired vision in the poor eye (45.3%, 34/75). Based on the available clinical data, the prevalence of strabismus in this cohort was 13.8% (9/65).

In the 75 probands, the highly myopic eyes had a mean spherical equivalent refraction of − 10.20 ± 3.30 diopters (median − 9.75 diopters, IQR − 11.75 to − 8.0 diopters) while the fellow eyes had a mean spherical equivalent refraction of − 0.70 ± 1.88 diopters (median − 0.25 diopters, IQR − 2.06 to 0.75 diopters). A difference of at least − 5.00 diopters was observed in all patients (Fig. 1C, D). Consistent with this, the mean total axial length of the highly myopic eye was 26.18 ± 1.70 mm (median 25.87 mm, IQR 25.26 to 26.98 mm) while that of the fellow eye was 22.90 ± 1.09 mm (median 22.75 mm, IQR 22.25 to 23.73 mm) (Fig. 1A). The relationship between the intraocular difference in axial length and the intraocular difference in refractive error is shown in Fig. 1B.

The intraocular difference in axial length in these patients is consistent with the intraocular difference in refraction error (R^2^ = 0.79, *P* < 0.0001) (Fig. 1B), based on correlation analysis of 60 of 75 probands with both data of refraction and axial length, in which the average axial length difference between the highly myopic eye and the fellow eye was 3.27 ± 1.31 mm and the average refractive error between the highly myopic eye and the fellow eye was 9.30 ± 3.31 diopters. The linear regression coefficient of the relationship was 0.3505, indicating that each 1 mm intraocular difference in axial length corresponds to the intraocular difference of 2.81 diopters.

**Fig. 2:**
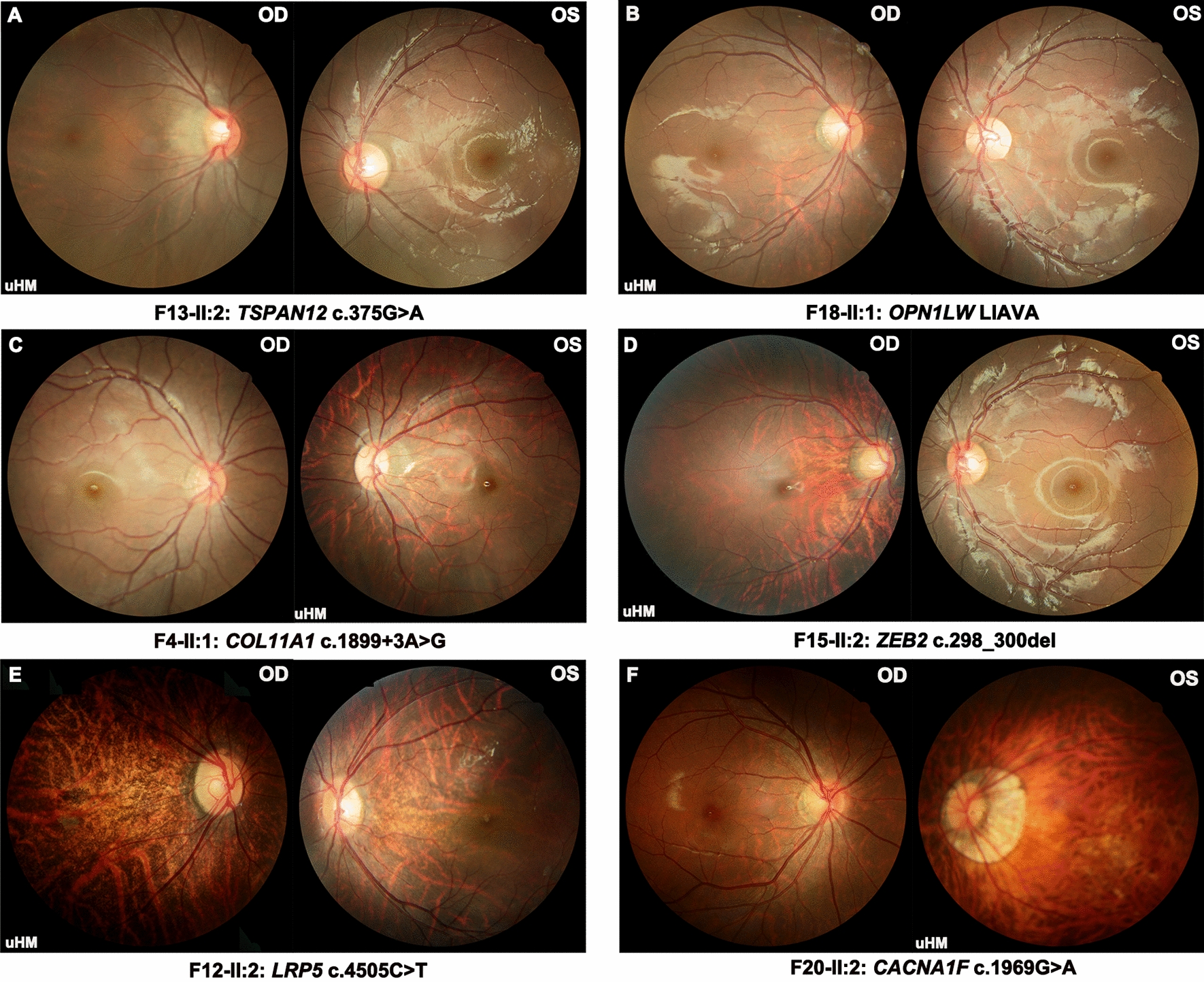
The posterior fundus photographs of bilateral eyes of six patients in this cohort. The uHM presents unilateral high myopia. The uHM symbol is in the lower-left corner of the fundus image of the highly myopic eye. The traditional fundus images of six patients show typical myopic fundus. **A**–**C** the fundus images of three probands (F13-II:2; F18-II:1; F4-II:1) reveal the tessellated fundus (C1) in the highly myopia eye and normal appearance (C0) in the fellow eye. **D** the fundus image of the proband (F15-II:2) shows diffuse chorioretinal atrophy (C2) in the highly myopic eye and normal appearance in the fellow eye. **E** the fundus images of the proband (F12-II:2) shows diffuse chorioretinal atrophy (C2) in both eyes. **F** the fundus photography of the proband (F20-II:2) demonstrates the macular atrophy (C4) in the highly myopic eye and the tessellated fundus (C1) in the fellow eye

According to the myopic maculopathy classification system [26], a tessellated fundus (Category 1), diffuse chorioretinal atrophy (Category 2), and macular atrophy (Category 4) were observed in 77.3% (58/75), 20.0% (15/75), and 2.7% (2/75) of patients, respectively (Fig. 1A). The available posterior fundus photographs revealed a normal-appearing or typical myopic appearance in the 75 probands (Fig. 2). However, extensive ocular examinations including SLO, OCT, FFA, and ERG, ultra-widefield fundus photography and FFA images revealed specific fundus changes in 48% (36/75) of these probands (Additional file 1: Table S2). The distribution of peripheral fundus changes in the probands were demonstrated in the Additional file 1: Table S3-1. Of the 63 eyes in 36 probands with peripheral retinal changes, the most common abnormality was the peripheral retinal avascular zone (74.6%, 47/63), followed by neovascularization (54.0%, 34/63), fluorescein leakage (31.7%, 20/63), peripheral pigmentary changes (31.7%, 20/63), peripheral retinal degeneration (30.2%, 19/63), and followed by several others (Additional file 1: Table S3-1). In addition, there was no significant difference in the distribution of peripheral retinal degeneration between the highly myopic eye (36/75) and the fellow eye (27/75) (*Fisher’s exact* test, *P* = 0.558).

Available OCT findings demonstrate Grade 1 to Grade 2 foveal hypoplasia in eight probands and normal structures in 36 probands. Similarly, the available ERG examination results of 18 eyes from nine probands exhibited normal cone response in six eyes and normal rod response in nine eyes. The remaining eyes demonstrated a mild reduction in cone response in four eyes, moderate reduction in one eye, and severe reduction in seven eyes. Additionally, there was a mild reduction in rod response in five eyes, moderate reduction in one eye, and severe reduction in three eyes (Additional file 2: Fig. S1).

### Molecular findings

**Fig. 3:**
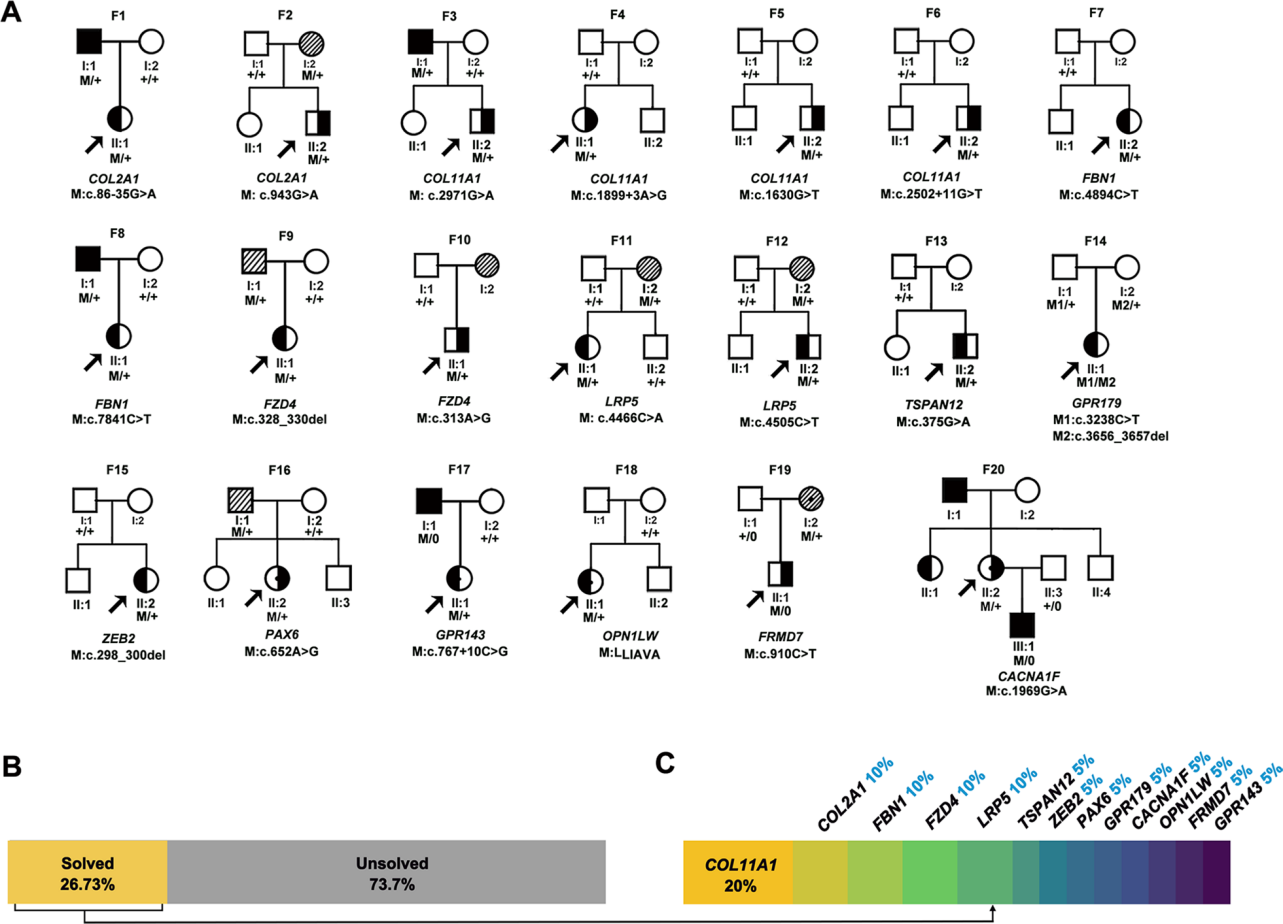
The molecular testing results and genetic landscape of the unilateral high myopia cohort. **A** Pedigrees of 20 unilateral families with potential pathogenic variants in 13 genes. The phenotypes of the patients shown in the pedigrees were based on the first visit. A solid black pattern indicates high myopia, a half-filled pattern indicated unilateral high myopia. a striped pattern indicates mild to moderate myopia. Circles represent females, squares represent males, and circles with a dot in center indicate heterozygous female carriers. M indicates mutant allele, + indicates the normal allele. Black arrows point to probands. **B** The molecular test results of 75 unrelated pedigrees with unilateral high myopia. Potential pathogenic variants in 13 genes were identified in 26.73% of the in-house cohort (20/75). **C** The distribution of the contributions of 13 genes to unilateral high myopia in these patients

The results of the genetic screening of the 75 probands with uHM from unrelated families are shown in Additional file 1: Table S2. After the detected variants were systematically evaluated with reference to the ACMG/AMP criteria, 21 potential pathogenic variants in 13 genes were identified in 20 of 75 (26.67%) probands, including two in-frame variants, nine truncation variants, and ten missense variants (Fig. 3 and Table 1; Additional file 1: Table S2). These variants in the 20 probands could be classified as pathogenic (6 probands), likely pathogenic (6), and variant of uncertain significance (9) based on ACMG/AMP criteria (Table 1). In addition, these variants are predicted to be deleterious by at least five in-silico prediction tools or were predicted to have an impact on splicing sites, as determined by the HSF tool. Finally, the majority of these variants (15/21) were absent in the gnomAD database, while the other four variants were extremely rare, with allele frequencies below 0.0001 and  the last two were compound heterozygous variants in *GPR179*. The 21 variants were confirmed by the Sanger sequencing (Additional file 2: Fig. S2).

**Table 1:** Rare variants in patients with unilateral high myopia

Variants	Family	Gene (ReIseqID)	Nucleotide acid	Amino acid	REVEL	CADD	①	②	③	④	⑤	HSF	ACMG-AMP
	ID		Change	Effect									Criteria
A. Pathogenic variants													
1	F13-II:2	TSPAN12 (NM_012338.3)	c.375G > A	p.Trp125*	/	/	/	/	/	/	DM	/	PVS1;PS1;PM2;PM4;PM6;PP4
2	F15-II:2	*ZEB2* (NM_014795.4)	c.298_300del	p.Asn100del	/	/	/	/	/	/	DM	NSSC	PS1;PS2;PM2;PM4;PM6
3	F14-II:1	*GPR179* (NM_001004334.4)	c.3238C > T	p.Gln1080*	/	/	/	/	/	17/280534	DM?	/	PVS1; PM3;PM4; PP1
4	F14-II:1	*GPR179* (NM_001004334.4)	c.3656_3657del	p.Pro1219Argfs*18	/	/	/	/	/	51/280988	DM?	/	PVS1;PM3;PP1
5	F18-II:1	*OPN1LW* (NM_020061.6)	L_LIAVA_	/	/	/	/	/	/	/	DM	SSC	PVS1;PS1;PS2; PS3;PM2;PP1
6	F19-II:1	*FRMD7* (NM_194277.2)	c.910C > T	p.Arg304*	/	/	/	/	/	1/183050	DM	/	PVS1;PS1;PM2;PM4;PP1;PP4
B. Likely pathogenic variants													
1	F2-II:2	*COL2A1* (NM_001844.4)	c.943G > A	p.Gly315Ser	0.97	28.8	D	PD	D	/	/	SSC	PM5;PM2;PP1;PP3
2	F3-II:2	*COL11A1* (NM_001854.4)	c.2971G > A	p.Gly991Ser	0.982	31	D	PD	D	/	/	SSC	PM5;PM2;PP1;PP3;PP4
3	F7-II:2	*FBN1* (NM_000138.4)	c.4894C > T	p.Arg1632Cys	0.602	32	D	PD	D	1/251312	/	NSSC	PS2;PM5;PM2;PP3
4	F9-II:1	*FZD4* (NM_012193.3)	c.328_330del	p.Ile110del	/	/	/	/	/	/	/	SSC	PM4;PM2;PP1;PP3;PP4
5	F10-II:1	*FZD4* (NM_012193.3)	c.313A > G	p.Met105Val	0.511	24.9	T	PD	N	6/250246	DM	SSC	PS1;PP1;PP3;PP4
6	F11-II:1	*LRP5* (NM_002335.3)	c.4466C > A	p.Thr1489Lys	0.751	26.7	D	PB	D	/	/	SSC	PM5;PM2;PP1;PP3;PP4
C. Uncertain significance variants													
1	F1-II:1	*COL2A1* (NM_001844.4)	c.86-35G > A	/	/	/	/	/	/	/	/	SSC	PM2;PP1;PP3;PP4
2	F4-II:1	*COL11A1* (NM_001854.4)	c.1899 + 3A > G	/	/	/	/	/	/	/	/	SSC	PM6;PP3;PP4
3	F5-II:2	*COL11A1* (NM_001854.4)	c.1630G > T	p.Gly544Trp	0.95	33	D	PD	D	/	/	NSSC	PM2;PP1;PP3
4	F6-II:2	*COL11A1* (NM_001854.4)	c.2502 + 11G > T	/	/	/	/	/	/	/	/	NSSC	PM2;PP1
5	F8-II:1	*FBN1* (NM_000138.4)	c.7841C > T	p.Ala2614Val	0.434	23.2	T	PD	N	/	/	NSSC	PM2;PP1
6	F12-II:2	*LRP5* (NM_002335.3)	c.4505C > T	p.Pro1502Leu	0.743	29.4	D	PD	D	/	/	NSSC	PM2;PP1;PP3;PP4
7	F16-II:2	*PAX6* (NM_000280.5)	c.652A > G	p.Thr218Ala	0.906	23.5	D	PD	D	/	/	NSSC	PM2;PP1;PP3;PP4
8	F17-II:1	*GPR143* (NM_000273.2)	c.767 + 10C > G	/	/	/	/	/	/	/	/	NSSC	PM2;PP4
9	F20-II:2	*CACNA1F* (NM_005183.4)	c.1969G > A	p.Ala657Thr	0.749	24.5	D	PB	D	12/168126	/	NSSC	PM2: PP1; PP3

For *COL11A1* in gnomAD, 5% variants had REVEL or CADD scores greater than 0.867 or 32, while 75% had such scores less than 0.657 or 27.2. For *COL2A1* in gnomAD, 5% variants had REVEL or CADD scores greater than 0.600 or 29.6, while 75% had such scores less than 0.311 or 27.4. For *LRP5* in gnomAD, 5% variants had REVEL or CADD scores greater than 0.940 or 31, while 75% had such scores less than 0.740 or 25.1. For *FZD4* in gnomAD, 5% variants had REVEL or CADD scores greater than 0.787 or 28.6, while 75% had such scores less than 0.613 or 24.2. For *FBN1* in gnomAD, 5% variants had REVEL or CADD scores greater than 0.863 or 29, while 75% had such scores less than 0.665 or 25.6. For *CACNA1F* in gnomAD, 5% variants had REVEL or CADD scores greater than 0.909 or 26.8, while 75% had such scores less than 0.845 or 25.9. For *PAX6* in gnomAD, 5% variants had REVEL or CADD scores greater than 0.880 or 29.9, while 75% had such scores less than 0.610 or 24.8

*PD* Probably damaging, *PB* Possibly damaging, *D* Damaging, *B* Benign, *T* Tolerant, *N* Neutral, *DM* Damaging mutation, */* Not available, *SSC* Splicing sites changes, *NSSC* No significant impact on splicing site. *①* SIFT, *②* Polyphen-2, *③* PROVEAN, *④* GnomAD allele frequency, *⑤* HGMD database

Of the 13 genes, variants in genes related to Stickler syndrome were most common, including *COL11A1* (4/20, 20%) and *COL2A1* (2/20, 10%). Variants in genes associated with FEVR were the second leading causative factor in solved uHM probands: *FZD4* (2/20, 10%), *LRP5* (2/20, 10%), and *TSPAN12* (1/20, 5%). The variants in the remaining 9 probands were detected in the following genes, which contribute to other related diseases when mutated, including *FBN1, GPR179, ZEB2*, *PAX6, GPR143*, *OPN1LW, FRMD7,* and *CACNA1F* (Fig. 3C). Interestingly, three female probands with uHM had heterozygous variants in X-linked genes (*GPR143*, *OPN1LW,* and *CACNA1F*), suggesting a skewed inactivation of the X chromosome between their two eyes.

### Genotype–phenotype correlation of uHM probands

The 20 probands with genetic variants identified in known myopia-related genes presented as simplex uHM without any other ocular conditions on their first visit to the general eye clinic. Additionally, while most parents only had mild to moderate myopia, so that the initial diagnosis of the 20 probands was presumed to be simplex uHM based on routine ocular examination (Fig. 2 and Table 2). There was no difference in population characteristics, including age, gender, visual acuity, refraction error, and axial length between the 20 probands with identified genetic defects and the remaining 55 probands without genetic variants (Additional file 1: Table S4). However, the myopic maculopathy in the highly myopic eye tended to be more severe in these 20 probands compared to the remaining probands (*Fisher’s exact test, P* = 0.003).

**Table 2:** Clinical phenotypes of 20 unilateral high myopia probands with molecular diagnostic testing

Family	Sex	Age		First	VA (Snellen)		Refraction (SER)		AL (mm)		Fundus	OCT		FFA		Diagnosis information	
ID		At onset	At exam	Symptom	OD	OS	OD	OS	OD	OS	OD	OS		OD	OS	Initial	Final
F1-II:1	F	3y4m	3y4m	PV on PE	0.1	0.2	− 9.5	− 2	23.78	21.95	TF;DCA;PS;PCC	N;	G1(OD)	/	/	uHM	STL
F2-II:2	M	5y10m	5y11m	PV on PE	1	0.05	0.75	− 14.75	23.1	29.22	N	TF;mild PCA;PCC	N	/	/	uHM	STL
F3-II:2	M	3y6m	4y7m	HM on PE	0.7	0.5	− 4.5	− 10.5	25.67	27.51	N	TF;DCA	/	/	/	uHM	STL
F4-II:1	F	3y3m	8y10m	PV on PE	0.6	0.3	− 5.75	− 13.75	24.96	27.93	N	TF;PCC	N	/	/	uHM	STL
F5-II:2	M	5y3m	5y5m	HM on PE	1	0.15	0.25	− 11.5	22.4	26.98	N	TF	N	/	/	uHM	STL
F6-II:2	M	5y2m	5y2m	HM on PE	0.8	0.04	1.5	− 12.25	22.68	27.83	N	FVH;TF;DCA;LD;PCD	G1(OS)	N	AZ;NV	uHM	STL
F7-II:2	F	5y4m	5y4m	PV on PE	FC	0.7	− 10	2	24.58	20.95	TF;RVs	N	N	/	/	uHM	MFS
F8-II:1	F	2y7m	3y7m	HM on PE	0.3	0.6	− 6.5	0	NA	NA	TF;PPA	N	/	/	/	uHM	MFS?
F9-II:1	F	EC	11y5m	Extropia	0.12	0.8	NA	NA	27.28	23.42	TF;DCA	N		AZ;NV;FL	AZ;NV;FL	uHM	FEVR
F10-II:1	M	2y9m	4y9m	MYP	0.5	0.4	− 2.75	− 10	24.54	27.38	TF;CC;LD	ODH;TF;DCA;LD;PCD	/	AZ;NV	AZ;NV	uHM	FEVR
F11-II:1	F	5y	6y1m	MYP	0.4	0.8	− 10	0.25	25.66	22.26	TF;Rvs;PCD	N;PCD	N	AZ;NV;FL	AZ;NV;FL	uHM	FEVR
F12-II:2	M	4y11m	5y3m	MYP	0.1	0.4	− 11.5	− 3.75	26.93	24.05	TF;DCA;PS;LD	TF;DCA;PS;PCD	G1(OU)	/	/	uHM	FEVR
F13-II:2	M	10m	4y10m	Photophobia	0.03	0.8	− 6	1.65	25.87	22.64	TF; ODH*	N	N	ME;AZ;NV;FL	AZ;NV;FL	uHM	FEVR
F14-II:1	F	1y4m	3y4m	HM on PE	0.2	0.4	− 8.75	− 2.5	24.38	22.45	TF;PPA:PCC	TF;PPA;PCC	/	/	/	uHM	CSNB1E
F15-II:2	F	6y3m	6y3m	PV on PE	0.03	1	− 12.25	− 0.25	NA	NA	TF;PPA;DCA	N	/	Hypo;Hype-AF^#^	Hyper-AF^#^	uHM	uHM
F16-II:2	F	7y8m	7y11m	PV on PE	1	0.1	0.5	− 8.5	23.45	26.82	TF;ODH;PPA	TF;DCA	N	/	/	uHM	uHM
F17-II:1	F	3y	4y4m	PV on PE	0.4	0.7	− 6	0.25	23.53	21.71	TF;PPA;PCC	PCC	N	/	/	uHM	OA1 carrier
F18-II:1	F	2y3m	4y3m	MYP	0.2	0.6	− 11.5	− 1	25.25	21.96	TF;PPA	N	G1(OU)	/	/	uHM	uHM
F19-II:1	M	4y6m	4y6m	EC-NYS	0.12	0.01	0.5	− 9	22.9	25.86	TF;FVH	TF;DCA;FVH	/	/	/	uHM	NYS1
F20-II:2	F	un	29y	HM on PE	0.5	HM^#^	− 4.75	− 15.12	24.13	30.2	TF; PPA	TF;PA;MA	N	/	/	uHM	CSNB2A carrier

*FFA* fundus fluorescein angiography, *OCT* Optical coherence tomography, *ERG* The electroretinogram, *VA* Visual acuity, *SER* Spherical equivalent refractive errors, *M* Male, *F* Female;, *N* Normal, *NA* Not available, *PE* Poor eye, *PV* Poor vision, *HM*^*#*^ Hand movement, *FC* Finger counting, *HM* high myopia, *TF* Tessellated fundus, *PPA* Myopic peripapillary crescent, *PS* Posterior staphyloma, *DCA* Diffuse chorioretinal atrophy, *MA* Macular atrophy, *PA* Patchy atrophty, *RVs* Retinal vessels straightening, *FVH* Foveal hypoplasia, *LD* Lattice degeneration, *ODH* Optic disc hypoplasia, *ODT* Optic disc tilt, *PCD* Peripheral retinal degeneration, *PCC* Peripheral retinal pigmentary change, *AZ* Avascular zone, *NV* Neovascularization, *FL* Fluorescein leakage, *ME* Macular ectopia, *CC* Chorioretinal coloboma, *uHM* unilateral high myopia, *STL* Stickler syndrome, *MFS* Marfan syndrome, *CSNB1E* complete congenital stationary night blindness, *CSNB2A* incomplete congenital stationary night blindness 2A, *OA1* ocular albinism type I; *NYS1* infantile nystagmus

^#^The FFA images of the proband (F15-II:2) showed hypofluorescence in the entire crescent area of the right eye and hyperflourensce in the optic disc area of both eye

**Fig. 4:**
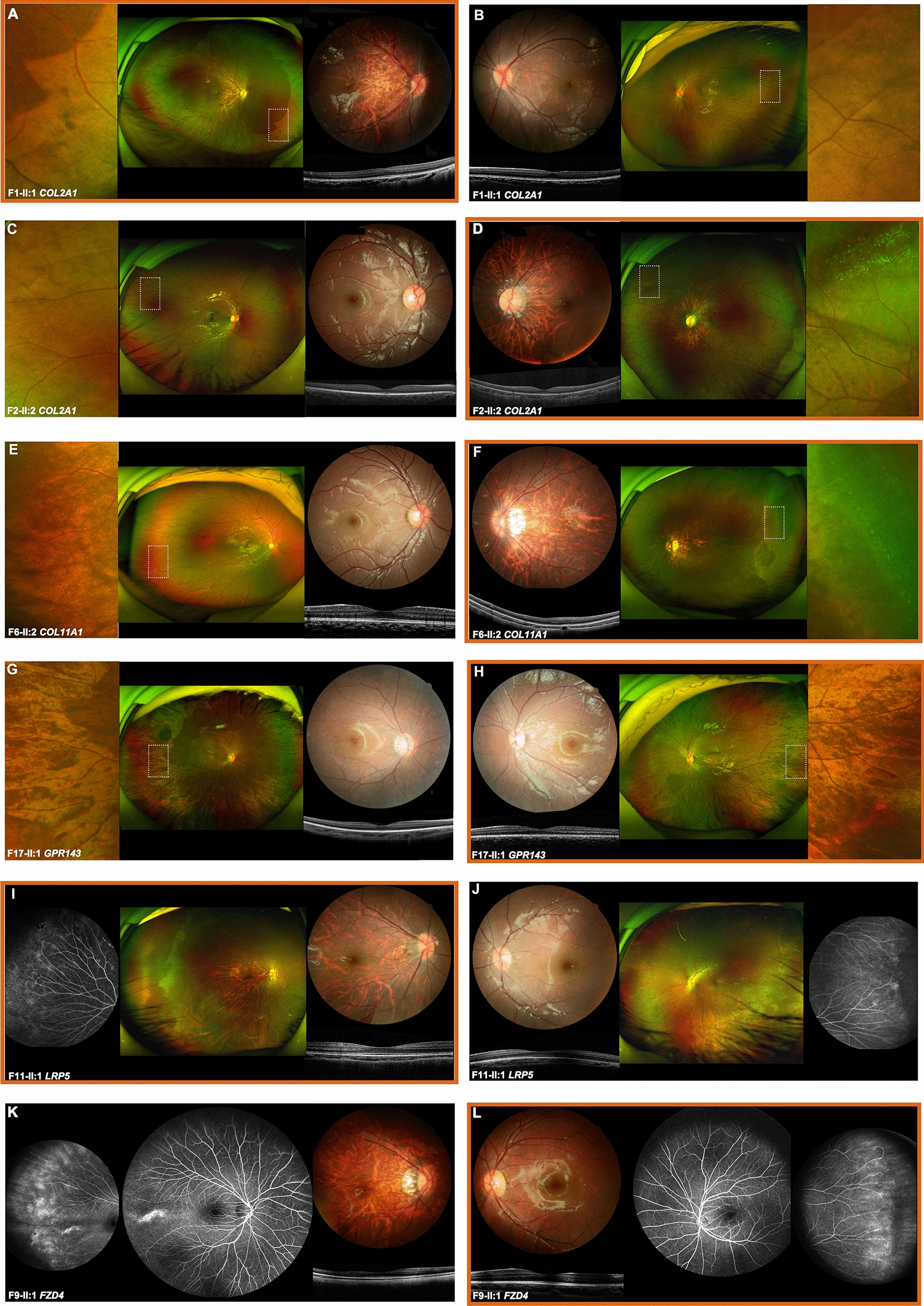
The specific ophthalmology examination results during follow-up for six probands in our cohort. The clinical findings of highly myopic eyes are boxed in red. **A**–**F**, the traditional fundus images of the three patients (F1-II:1; F2-II:2; F6-II:2) with variants in genes related to Stickler syndrome (*COL2A1* and *COL11A1*) demonstrates the typical myopic fundus, while ultra-widefield fundus images show the specific peripheral fundus changes including lattice degeneration and peripheral retinal pigmentation in the highly myopic eyes. The corresponding magnified images of the local retinal region (outlined by white dashed lines in an ultra-widefield fundus image) illustrate the similar fundus changes. **G**–**H**, the posterior fundus images of the *GPR143* carrier (F18-II:1) shows myopic fundus, and ultra-widefield fundus images and magnified images demonstrate the nonuniform fundus pigmentation in both eyes. I-L, the fundus images of two probands (F11-II:1; F9-II:1) with variants in genes related to FEVR (*LRP5* and *FZD4*) illustrate classic myopic fundus in the posterior pole, the peripheral avascular region, and fluorescein leakage were observed in fluorescein angiography

Extensive ophthalmological evaluation identified peripheral specific signs in these patients, corresponding to their genetic findings. Among these peripheral retinal changes, variants in genes related to Stickler syndrome were predominantly associated with peripheral retinal pigmentary changes (50%), lattice degeneration (25%), or peripheral avascular region (25%) in this cohort (Additional file 1: Table S3-2). For example, the peripheral fundus images of proband with variants in genes related to Stickler syndrome show lattice degeneration or peripheral retinal degeneration in the highly myopic eye only, and peripheral retinal pigmentary change in both eyes (Fig. 4A–F).

On the other hand, the variants in genes related to FEVR were mainly associated with peripheral retinal avascular zone (100%), neovascularization (90%), and fluorescein leakage (60%) (Additional file 1: Table S3-2). The peripheral retinal degeneration was also found in both eyes of all patients with variants in *LRP5* (Fig. 4I–J). The FFA images of proband F9-II:1 demonstrate bilateral peripheral avascular areas, fluorescein leakage, and neovascularization, despite the myopic or normal appearance in the posterior fundus, consistent with FEVR disease caused by the *FZD4 v*ariant c.328_330del (p.Ile110del) (Fig. 4K, L). A similar pattern of fundus changes was also observed in proband F11-II:1 with *LRP5* variant 4466C > A (p.Thr1489Lys) (Fig. 4I, J). In comparison to the fellow eye, patients with uHM and FEVR tended to have a more advanced stage of FEVR in the highly myopic eye (*P* = 0.043) (Fig. 1A).

Moreover, the SLO images of *GPR143* c.767 + 10C > G carrier (F17-II:1) showed bilateral mosaic fundus pigmentation with radially hypopigmentation streaks oriented in the peripheral region with a normal-like posterior pole consistent with the specific changes in OA1 carriers (Fig. 4G, H) in whom uHM is relatively rare. For the variants in other genes such as *FBN1*, *ZEB2*, *GPR179*, *PAX6, OPN1LW*, *FRMD7*, and *CACNA1F*, specific peripheral changes were relatively rare in probands carrying variants in these genes, which was indeed aligning with the genetic findings. In summary, the final diagnostic phenotypes of the 20 probands or their affected family members were based on genetic variants as well as comprehensive ocular ophthalmic examinations, especially the strong correlation between the varied gene and closely related specific phenotypes. However, for those probands with unspecific retinal changes without identified genetic variants, a final diagnosis rather than uHM would be difficult in clinic (Table 2; Additional file 1: Table S2; Additional file 2: Fig. S2).

### Improvement of visual acuity in probands with uHM

**Fig. 5:**
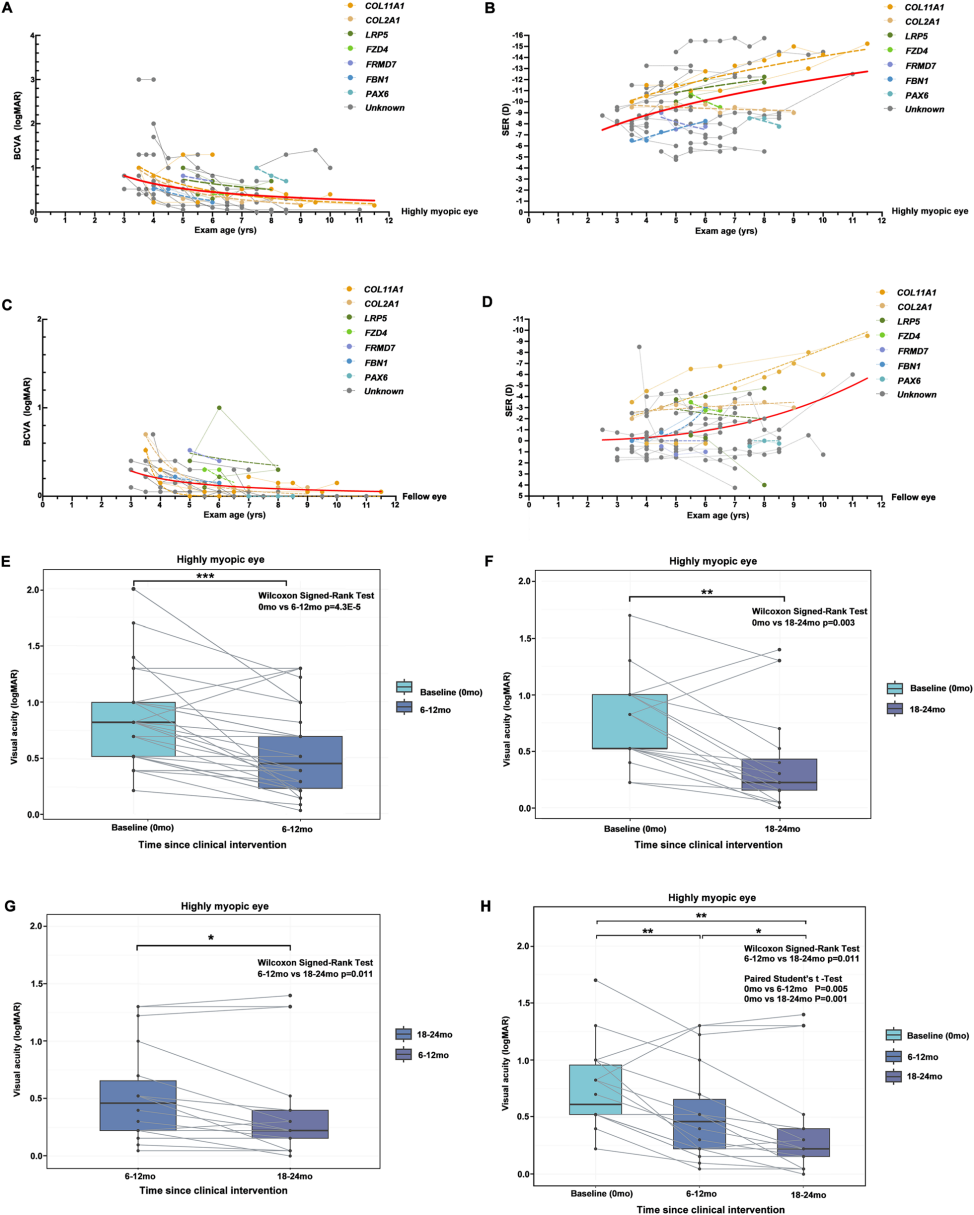
Best-corrected visual acuity and refraction progression of unilateral high myopia. Different colored points represent different genes, gray points represent patients without an identified genetic cause, and the red curve represents the best nonlinear fit of all data points. Scatter plots show the steady improvement of best-corrected visual acuity with age in the highly myopic eye (**A**) and the fellow eye (**C**). The other scatter plots show the gradual progression of refraction with age in the highly myopic eye (**B**) and the fellow eye (**D**). **E** Paired box plot showing the mean visual acuity of highly myopic eye improving from 0.84 to 0.56 logMAR (*P* = 4.3E−5) with clinical intervention over 6–12 months (38 patients). **F** Mean visual acuity of highly myopic eyes improved from 0.72 to 0.39 logMAR (*P* = 0.003) with clinical intervention in the period from 18 to 24 months (20 patients). **G**. Mean visual acuity improved from 0.53 to 0.40 with clinical intervention (*P* = 0.011) over periods from 6 to 12 months and 18 to 24 months (18 patients). **H** Comparison between the bassline visual acuity and final visual acuity at time points of 6–12 months and 18–24 months after clinical intervention (18 patients). *BCVA* = best-corrected visual acuity, *yrs* = years, *mo* = month

The mean logMAR BCVA of the highly myopic eye and the fellow eye were 0.89 ± 0.63 (median 0.7, IQR 0.43 to 1.00) and 0.17 ± 0.19 (median 0.1, IQR 0.0 to 0.3), respectively. There was a statistically significant difference between the BCVA of the highly myopic eye and that of the fellow eye (*Wilcoxon signed-rank test,* P < 0.001). Based on results of the follow-up study, uHM probands demonstrated steady BCVA improvement and gradual progression of refractive error (Additional file 1: Table S5). There were similar BCVA improvements in both eyes of these probands (Fig. 5A, C), but different rates of refractive error progression were observed in the highly myopic eye and the fellow eye of uHM probands. Compared to the fellow eye, the highly myopic eye of uHM children had a higher progression rate of refraction error (Fig. 5B, D). According to the available follow-up study results for 38 uHM probands, the mean logMAR BCVA of the highly myopic eye improved from 0.84 to 0.56 within 6 to 12 months of clinical intervention (optical correction together with patch therapy) (*Wilcoxon signed-rank test*, *P* = 4.3E−5) (Fig. 5E). Similarly, based on the follow-up data available for 20 uHM probands, the mean logMAR BCVA of highly myopic eyes improved from 0.72 to 0.39 with clinical intervention over the period from 18 to 24 months (*Wilcoxon signed-rank test*,* P* = 0.003) (Fig. 5F). Moreover, the mean logMAR BCVA further improved from 0.53 to 0.40 with clinical intervention (*Wilcoxon signed-rank test*, *P* = 0.011) between the period 6 to 12 months and 18 to 24 months (Fig. 5G). However, it is worth noting that the BCVA of two probands (F5-II:1 and F44-II:1) had decreased slightly in 6–12 months, and for the one patient (F44-II:1), there was a slightly worsening from that point to 18–24 months (Fig. 5E–G). Additionally, the BCVA of other two patients (F43-II:1 and F47-II:1) improved within 6–12 months but slightly worsened from that time to 18–24 months (Additional file 1: Table S5 and Fig. 5F, H). The fluctuation of BCVA among these patients may be attributed to the poor compliance as no apparent change on posterior fundus was observed.

Consistent with the above results, follow-up of 18 uHM probands showed progressive improvement in the mean BCVA at each follow-up time point (baseline vs 6 to 12 months, *P* = 0.005; baseline vs 18 to 24 months, *P* = 0.001; 6 to 12 months vs 18 to 24 months, *P* = 0.011) (Fig. 5H). Among the 18 participants, at the end of 18 to 24 months, the BCVA of 15 (83.3%) uHM patients was 0.52 logMAR or better (Snellen fraction 20/63), and the BCVA of 11 (61.1%) uHM patients improved 3 or more lines.

## Discussion

In the current study, 75 probands with referring diagnosis of simplex uHM based on routine examination were recruited from our Pediatric and Genetic Eye Clinic. Extensive clinical and genetic analysis identified peripheral retinal changes with potentially severe consequences in 48% of probands with uHM while causative genetic defects were detected in 26.7% of them. These findings are of clinical significance not only for the complications with the potential to cause blindness in the patients themselves but also for genetic counseling for their siblings or offspring. As far as we know, our data present for the first time a systematic extensive clinical and genetic analysis of a cohort of uHM with important findings that would be valuable in improving the clinical care of patients with uHM.

Clinical care of uHM, complex or simplex, has been considered to be a challenge in the pediatric eye clinic, in which most attention has been paid to amblyopia and strabismus [4, 29]. Thus, uHM has been described as an accompanying sign associated with other ocular or systemic diseases in which the clinical signs of original diseases are remarkable, including corneal opacity [5], congenital lens abnormalities [2], uveal coloboma [6], morning glory syndrome [7], Straatsma syndrome [8], Stickler syndrome [9], familial exudative vitreoretinopathy [30], and retinopathy of prematurity [10], etc. Unlike the complex uHM cases mentioned above, simplex uHM patients usually lack noticeable suggestive signs of other ocular or systemic diseases except for a posterior myopic fundus on routine ocular examination. Such simplex uHM may mainly be treated as anisometropia alone with attention paid to amblyopia and strabismus in clinical practice [2, 4, 29]. Peripheral retinal changes underlying severe complications may be neglected because of an appearance of simple myopic fundus in uHM, especially when the OCT and ERG results reveal no significant abnormalities or only subtle changes, as they did in most of the probands enrolled in this study. In the current study, we detected obvious peripheral retinal changes including retinal degeneration, lattice degeneration, retinal pigmentary changes or hypopigmentation, peripheral avascular zone, neovascularization, fluorescein leakage, or fibrous proliferation in about half of the probands. In this study, the most common peripheral changes are peripheral avascular zone and neovascularization, which have been identified to be highly associated with variants in genes related to FEVR. Correspondingly, the peripheral retinal degeneration, lattice degeneration, and pigmentary changes have been observed in more than one-third of cases with peripheral retinal changes, highly associated with variants in genes related to Stickler syndrome. These findings indicate that the peripheral retinal changes may underlie vision-threatening diseases and emphasize the importance of systematic evaluation of the entire peripheral retina as soon as possible for children with uHM. Their importance is further shown by the detection in these patients of genetic variants in genes associated with ocular diseases frequently leading to blindness. Therefore, SLO or RetCam screening is recommended as an initial necessary examination for all patients with uHM. Additional specific examinations such as FFA or OCTA would be needed if suggestive signs were observed by SLO or RetCam. In addition, there is no statistically significant difference in the distribution of peripheral retinal changes between the highly myopic eye and the fellow eye, suggesting that the peripheral retinal examination should be performed not only in the highly myopic eye but also in the fellow eye.

There is a high correlation between axial length and spherical equivalent refraction in our patients, indicating that the asymmetric spherical equivalent refraction difference between both eyes in uHM patients can be primarily attributed to the interocular difference in total axial length, which is consistent with previous studies [2, 18, 31]. However, the contribution of other optical components, such as cornea and crystalline lens, cannot be ruled out. In this cohort, *FBN1* variants have been identified in two probands, associated with Marfan syndrome. Previous study suggests that the high myopia in Marfan syndrome may be derived from both lenticular as well as axial mechanisms [32]. Consistently, our recently published data also suggests that myopia in patients with Marfan syndrome initially presents as refractive myopia and gradually evolves into axial myopia [33]. This transition is attributed to the normal axial length in the early stages in these patients, which later develops into longer axial length with age and disease progression. Additionally, the minimal axial length difference in this cohort is 1.43 mm (contributed to about 4 diopter difference), observed in the proband F52-II:1. Despite an 8.5 diopter refraction difference in this patient, inconsistent with axial length difference, this might be partly explained by the young age of the proband (examined at 3 years old). Alternatively, it implies a potential contribution of other optical components to refractive asymmetry, as some studies propose that interocular difference in corneal or crystalline lens structure may contribute to the asymmetry in refractive errors. [18, 34–36]

There have been many genetic studies on bilateral eoHM in recent years and a number of genetic factors contributing to or associated with eoHM have been identified [12], so that currently genetic variants are seen in about one third of families with monogenic eoHM (about 24% in RetNet genes [13, 14] and 9% in genes for high myopia [15–17] based on our previous studies). However, genetic analysis of uHM has been lacking although most uHM is also early-onset, usually occurring in early childhood. In the current study, 21 potential pathogenic variants in 13 genes were identified in 26.7% (20/75) of probands by exome sequencing, with a detection rate comparable to that for eoHM, suggesting a similar genetic contribution to both uHM and eoHM. Similarly, genetic contributions have also been observed in patients with bilateral or unilateral microphthalmia or coloboma [37–39]. In previous studies, uHM has been suggested to be primarily influenced by genetic factors rather than environmental factors, especially when it occurs at a young age [2, 18], although exact genetic defects have not been explored. It remains puzzling how genetic defects lead to asymmetric ocular growth between the two eyes. In fact, the asymmetry is not a rare phenomenon in inherited ocular diseases. For the patients with microphthalmia, anophthalmia and coloboma (MAC), the asymmetric development of the two eyes is common [37, 40]. The genetic diagnostic rate of unilateral MAC patients has been found to be similar to that for bilateral MAC patients [37]. In addition, the unilateral retinal changes also have been found in FEVR patients, and causative genetic variants have been identified in more than half of unilateral FEVR patients [41]. Similarly, asymmetry can also be observed in Mendelian inherited systemic disorders such as Goldenhar syndrome [42, 43]. Nevertheless, the exact molecular mechanism for uHM remains unclear. This phenomenon might relate to changes in gene expression due to DNA methylation or other epigenetic control mechanism [44], or perhaps due to modifying genes, a environmental modifier, or developmental stochastic effect [45]. In this study, heterozygous variants in X linked genes, including *GPR143*, *OPN1LW*, or *CACNA1F*, have been identified in three probands. The skewed X-chromosome inactivation in different eyes might account for asymmetric ocular changes in the female patients [2]. Recently, *PAX6* and *ZFHX1B* variants were reported to be involved in developmental anisometropia through regulating the unbalanced elongation of axial length between the eyes [46]. Coincidentally, the variants in both *PAX6* and *ZFHX1B* (*ZEB2)* have been identified in uHM probands in this study [46]. The other detected genes in this study, such as those related to Stickler syndrome (*COL2A1* and *COL11A1) or* Marfan syndrome (*FBN1*), which have been proposed to modulate ocular growth through axial mechanism [32, 47]. However, myopia in FEVR has been suggested be through a lenticular mechanism, [32, 48], anisometropia has been observed as a common sign in patients with FEVR [41]. The exact mechanism underlying uHM or asymmetrical development of the eye is unclear [29, 49]. In general, the mechanisms through which genetic defects contribute to the development of uHM and unilateral diseases in general remain a puzzle that requires further studies to unravel in the future.

As genetic defects contribute to a significant portion of uHM, it might be important to provide genetic testing and counseling for patients with uHM during their initial visit. The most common genetic cause of uHM in this study is variants in genes related to Stickler syndrome and FEVR, indicating that uHM had high risk of retinal detachment. In addition, our data suggests that the presence of other underlying inherited ocular diseases might be under-ascertained in simplex uHM. As seen in proband F20-II:2 with uHM and heterozygous *CACNA1F* p. Ala657Thr variant, subsequent analysis of her son demonstrated the same variant in hemizygous status and congenital stationary night blindness (CSNB) with bilateral high myopia. Thus, genetic screening for uHM patients can provide solid information for genetic counseling not only for the patients themselves, but also for family members, and especially for female carriers with X-linked diseases.

The prevalence and severity of amblyopia has been suggested to be gradually increased with age and with the magnitude of anisometropia during early childhood [50]. Gabai and Zeppieri reported that the optimal treatment window of anisometropia appears to be between 3 and 7 years of age, as the impact of age on treatment effectiveness is minimal during this period, while the efficacy of treatment decreased after this age [51]. Thus, timely recognition and intervention of uHM in pre-school children is important. The basic treatment for uHM is wearing spectacles for optical correction in combination with patch therapy. Several studies have identified the efficiency of such basic treatment on amblyopia associated with uHM in children [4, 52]. In the current study, the BCVA of children with uHM improves through correction of refractive error together with occlusion, consistent with previous studies. Apart from such basic treatment, the posterior scleral reinforcement combined with patching therapy had been suggested to contribute to delaying the myopia progression and improving the visual acuity based on study on 16 preschool uHM children [53]. As nearly half of the uHM probands in the current cohort exhibited peripheral fundus changes underlying risky complication, therefore, surgical interventions for young uHM children should be treated with great caution, emphasizing the necessity of comprehensive preoperative examinations. Further research is essential to validate the benefits and disadvantages of surgical interventions for young uHM children in the future. In addition, low-concentration atropine [54] and orthokeratology treatment [55] might delay the myopia progression on the highly myopic eye in children with myopic anisometropia. However, it's noteworthy that such intervention on myopia is mainly based on low to moderate myopia, other than uHM as seen in the current study. The effectiveness of these interventions in children with uHM requires further studies.

Although the current study provides valuable novel findings, there are some limitations. First, as described above, the number of uHM participants enrolled in the follow-up study of the BCVA improvement in highly myopic eye is limited due to variable time of follow-up duration, lack of placebo controls, and compliance of intervention in children. Therefore, the treatment outcome of the current study should be taken with caution. The accurate assessment of the effectiveness of the clinical intervention for uHM patients requires prospective cohort studies in the future. Second, the variants related to Stickler syndrome or Marfan syndrome have been commonly identified in this cohort. Since all subjects are recruited in our Pediatric and Genetic Eye Clinic, the majority of patients in this cohort are children (the age at examination was 6.21 ± 4.70 years) so that some of the valuable examinations (like SLO, FFA) were not performed in all probands. Furthermore, some extra-ocular manifestations related to these syndromes are not obvious in early childhood, which may present or become more obvious in the teenager period [56], so that gene test on these patients in young childhood may provide an early warning marker in the care of these diseases. However, the transition of related clinical manifestations still needs to be further observed in the future.

## Conclusions

In summary, to the best of our knowledge, the current study represents the first systematic clinical and genetic analysis of simplex uHM, revealing not only a clear association between monogenic variants and uHM but also potential blinding diseases hidden under classic myopic fundus. These findings emphasize the importance of early extensive examination of peripheral retina in both eyes of uHM patients and of genetic counseling for family members based on genetic testing. Regular follow up visit is crucial not only for care of amblyopia but also for early detection of potential complications, which is the key for effective management of blindness consequence. In addition, this study may enrich our understanding of the molecular basis underlying uHM. The risky factors identified in the current study may also be valuable to be explored in other related eye diseases, such as anisometropia and amblyopia.

### Supplementary Information

**Additional file 1: Table S1.** The gene list of the ophthalmic targeted exome sequencing panel included 736 genes. **Table S2.** Clinical phenotype and molecular diagnosis of 75 patients with unilateral high myopia. **Table S3-1.** The distribution of peripheral fundus findings in unilateral high myopia children. **Table S3-2.** The distribution of peripheral fundus findings in 20 unilateral high myopia children with genetic defects. **Table S3-3.** The distribution of peripheral fundus findings in unilateral high myopia children with or without genetic defects. **Table S4.** Comparison of baseline characteristics between groups with and without genetic defects. **Table S5.** The follow-up study of refraction in patients with unilateral high myopia in this study.

**Additional file 2: Figure S1.** The optical coherence tomography (OCT) scans and the electroretinogram (ERG) recordings of eight unilateral high myopia probands in this cohort. **A**–**D** The OCT scans demonstrated normal structure in three eyes of two patients (F4-II:1 and F5-II:2). The grade 1 or grade 2 foveal hypoplasia was observed in five eyes of three patients (F1-II:1, F5-II:2, and F12-II:2). **E**-**H** The ERG findings of the highly myopic eye of four patients showed normal cone and rod response in two patients (F8-II:1 and F14-II:1), moderate to severe reduction in cone response, and severe reduction in rod response in the other two patients (F9-II:1 and F13-II:2). **Figure S2.** Family Pedigrees and Sanger sequencing chromatograms results for 20 unilateral high myopia families. The DNA sequencing results of affected patients and normal controls were presented in the right column. While the corresponding family pedigrees were displayed in the left column.

**Additional file 3: Figure S3.** Representative image of each peripheral retinal change identified in the study. **Table S6.** List of peripheral retinal changes in this cohort.

## Data Availability

All data generated during this study are available in the paper and its supplementary materials. The raw data used and/or analysed during the current study are available from the corresponding author on reasonable request.

## Abbreviations

uHM: Unilateral high myopia
FFA: Fundus fluorescein angiography
WES: Whole-exome sequencing
TES: Targeted exome-sequencing
SLO: Scanning-laser ophthalmology
OCT: Optical coherence tomography
ERG: Electroretinogram
eoHM: Early-onset high myopia
HGMD: Human Gene Mutation Database
HSF: Human splicing finder
ACMG/AMP: The American College of Medical Genetics and Genomics and the Association for Molecular Pathology
BCVA: Best corrected visual acuity
OCTA: Optical coherence tomography angiography
