# Supplementary material for: Clinical and genetic risk factors underlying severe consequence identified in 75 families with unilateral high myopia
Source: J Transl Med. 2024 Jan 19;22:75. doi: 10.1186/s12967-024-04886-5 (PMC10797748; doi:10.1186/s12967-024-04886-5)
Supplement: Supplementary file 2 — Additional file 2: Figure S1. The optical coherence tomography (OCT) scans and the electroretinogram (ERG) recordings of eight unilateral high myopia probands in this cohort. A–D The OCT scans demonstrated normal structure in three eyes of two patients (F4-II:1 and F5-II:2). The grade 1 or grade 2 foveal hypoplasia was observed in five eyes of three patients (F1-II:1, F5-II:2, and F12-II:2). E-H The ERG findings of the highly myopic eye of four patients showed normal cone and rod response in two patients (F8-II:1 and F14-II:1), moderate to severe reduction in cone response, and severe reduction in rod response in the other two patients (F9-II:1 and F13-II:2). Figure S2. Family Pedigrees and Sanger sequencing chromatograms results for 20 unilateral high myopia families. The DNA sequencing results of affected patients and normal controls were presented in the right column. While the corresponding family pedigrees were displayed in the left column. [file 12967_2024_4886_MOESM2_ESM.pdf]

Figure S1. The optical coherence tomography (OCT) scans and the electroretinogram (ERG) recordings of eight unilateral high myopia probands in this cohort. **(A-D)** The OCT scans demonstrated normal structure in three eyes of two patients (F4-II:1 and F5-II:2). The grade 1 or grade 2 foveal hypoplasia was observed in five eyes of three patients (F1-II:1, F5-II:1, and F12-II:2). **(E-H)** The ERG findings of the highly myopic eye of four patients showed normal cone and rod response in two patients (F8-II:1 and F14-II:1), moderate to severe reduction in cone response, and severe reduction in rod response in the other two patients (F9-II:1 and F13-II:2).

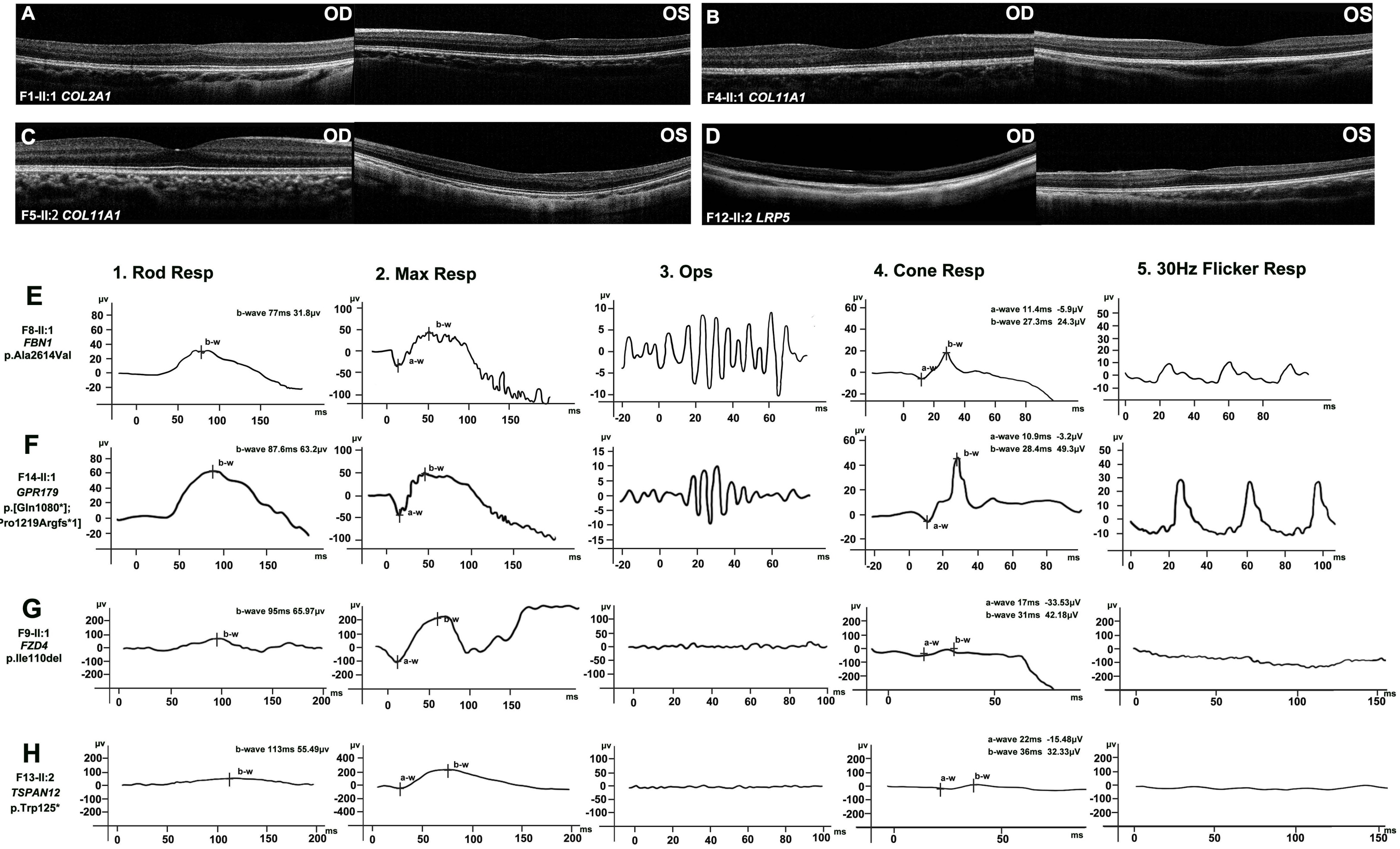

Figure S2. Family Pedigrees and Sanger sequencing chromatograms results for 20 unilateral high myopia families. The DNA sequencing results of affected patients and normal controls were presented in the right column. while the corresponding family pedigrees were displayed in the left column.

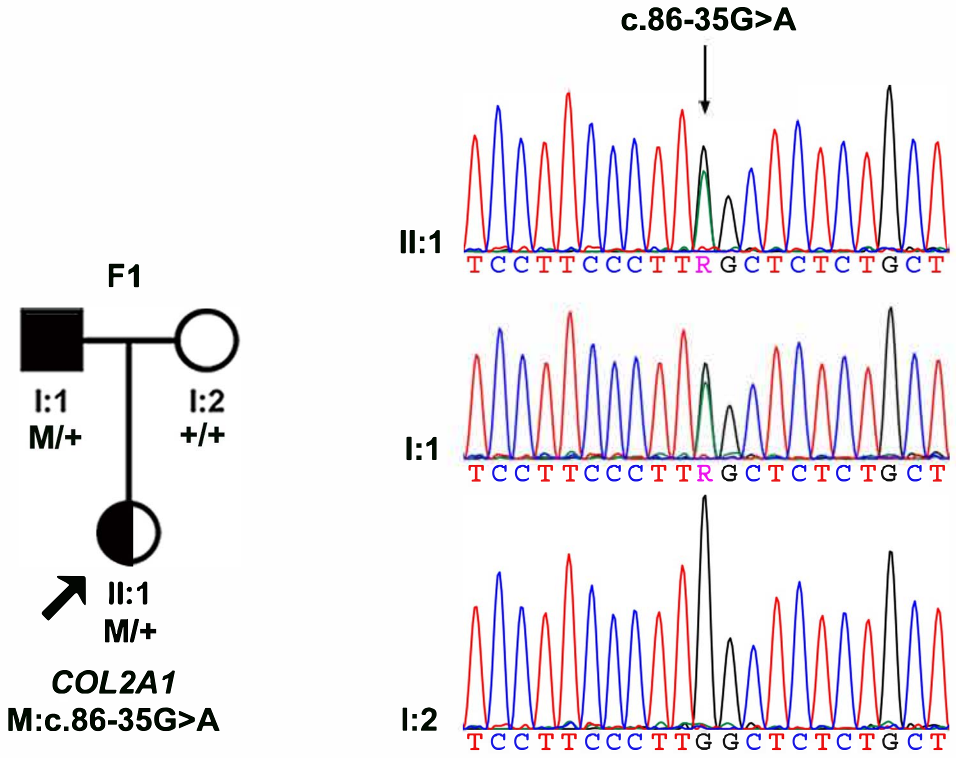

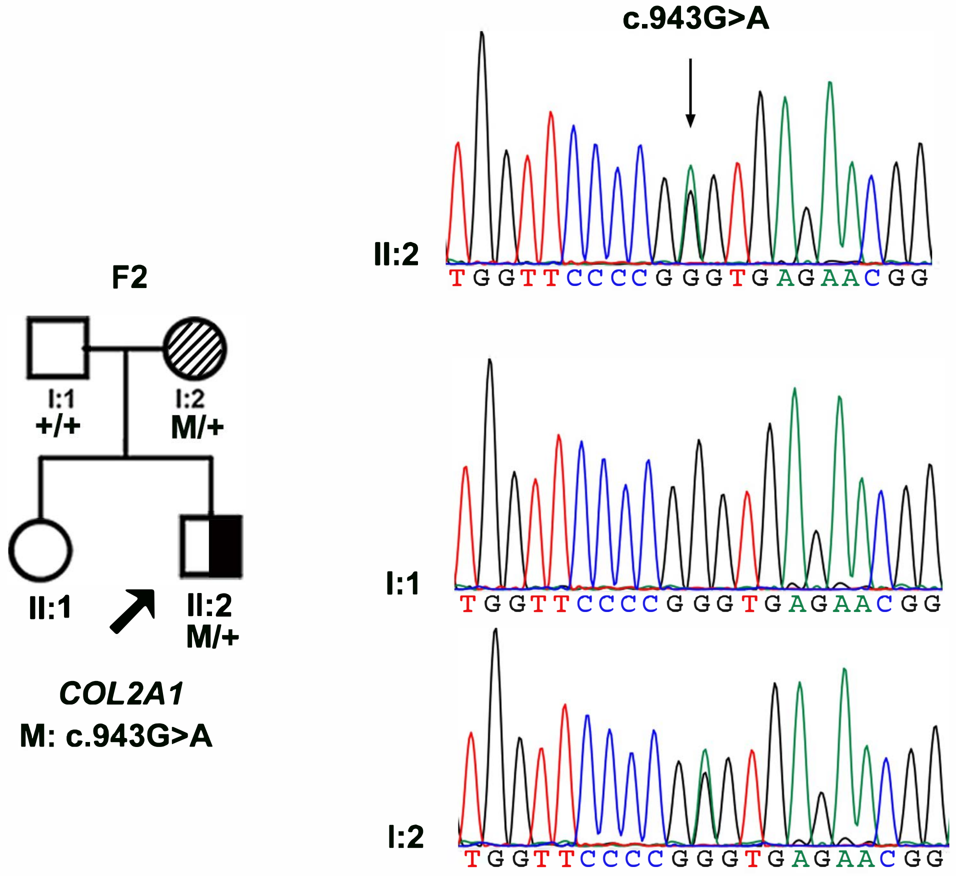

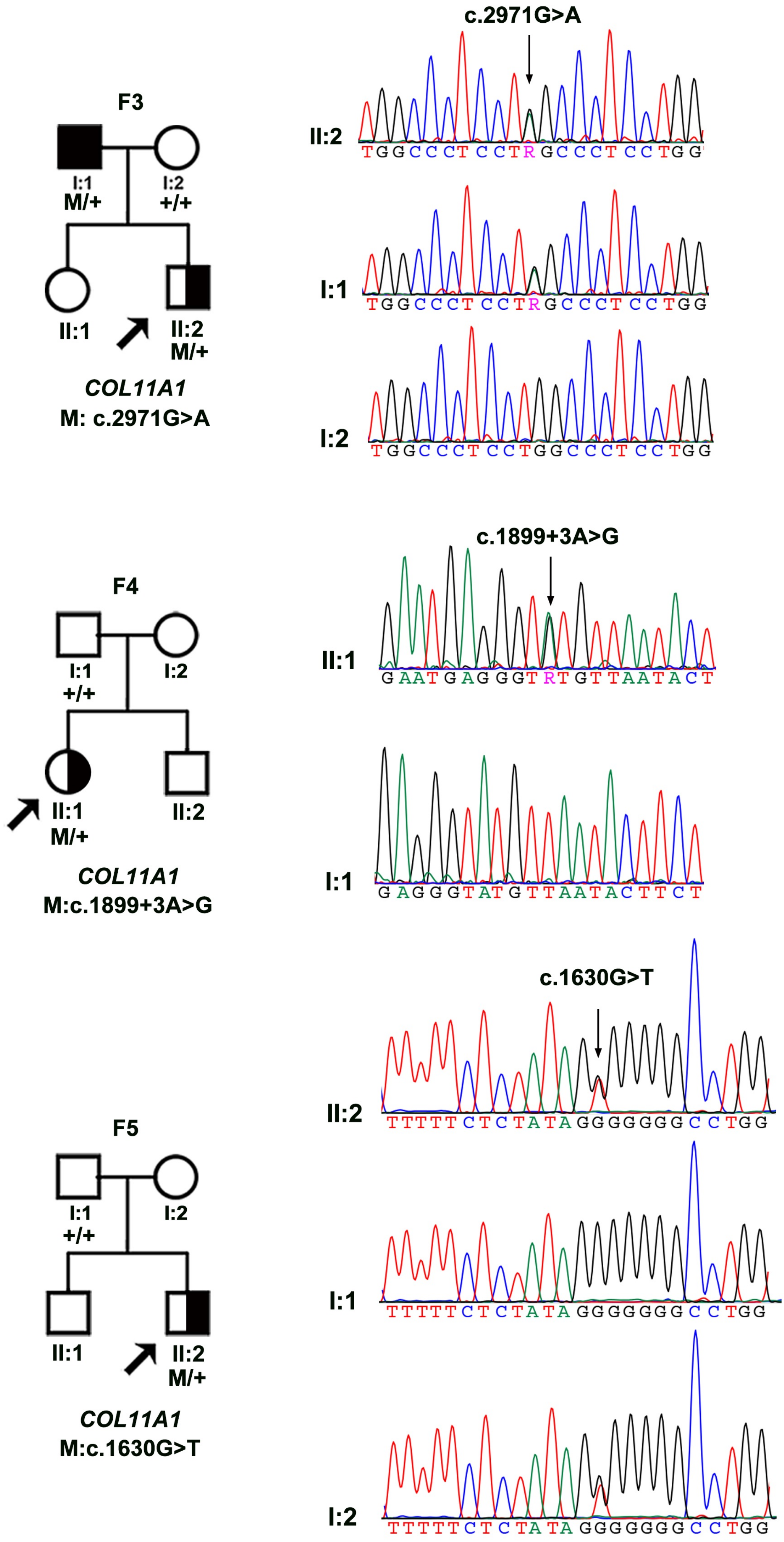

F3
I:1
M/+
I:2
+/+
II:1
II:2
M/+
COL11A1
M: c.2971G>A
c.2971G>A
II:2
TGGCCCTCCTRGCCCTCCTGG
I:1
TGGCCCTCCTRGCCCTCCTGG
I:2
TGGCCCTCCTGGCCCTCCTGG
F4
I:1
+/+
I:2
II:1
M/+
II:2
COL11A1
M:c.1899+3A>G
c.1899+3A>G
II:1
GAATGAGGGTRTGTTAATACT
I:1
GAGGGTATGTTAATACTTCT
F5
I:1
+/+
I:2
II:1
II:2
M/+
COL11A1
M:c.1630G>T
c.1630G>T
II:2
TTTTTCTCTATAGGGGGGCCTGG
I:1
TTTTTCTCTATAGGGGGGCCTGG
I:2
TTTTTCTCTATAGGGGGGCCTGG

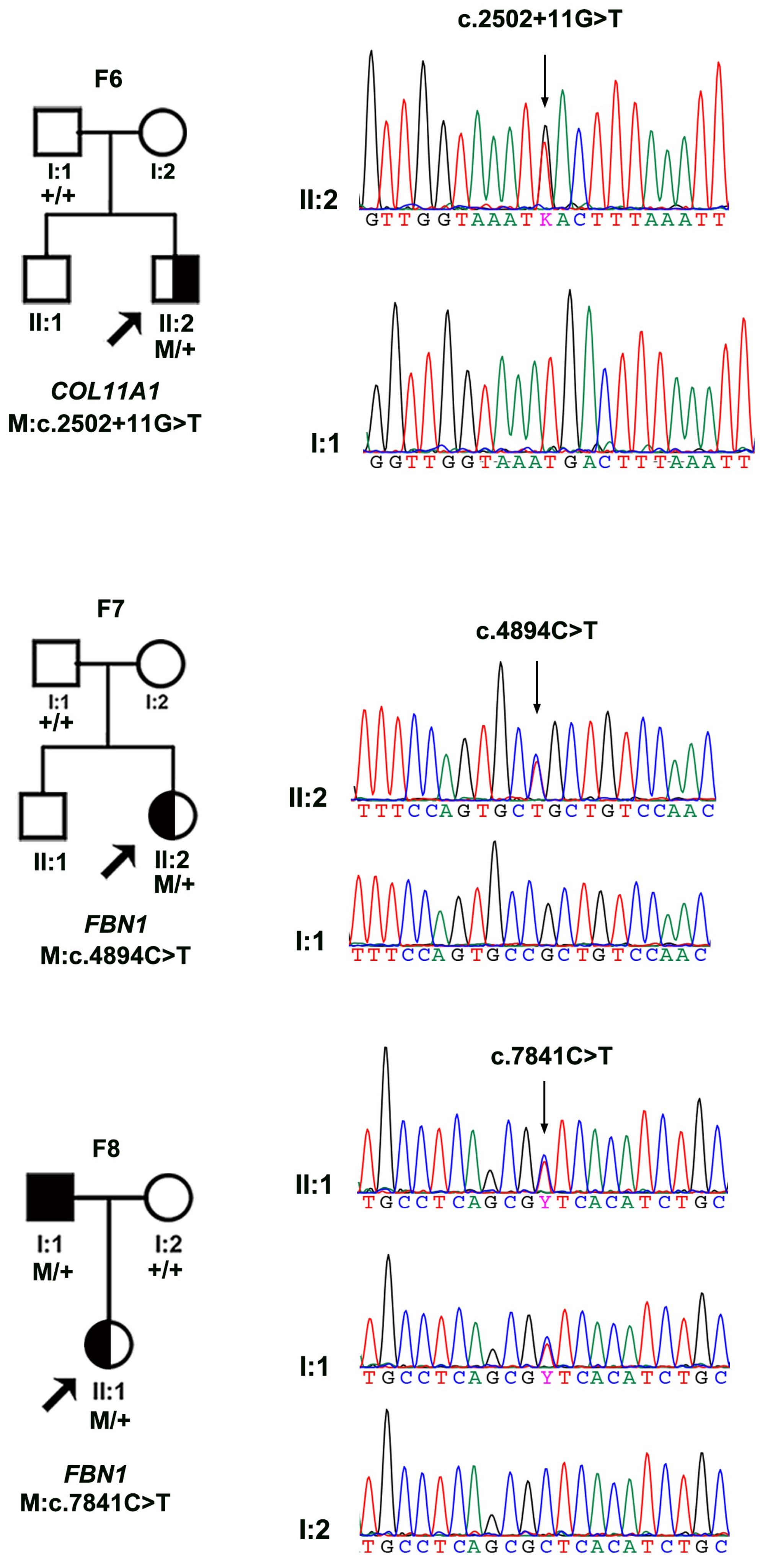
F6
I:1
+/+
I:2
II:1
II:2
M/+
COL11A1
M:c.2502+11G>T
c.2502+11G>T
II:2
GTTGGTAAATKACTTTAAATT
I:1
GGTTGGTAAATGACTTTAAATT
F7
I:1
+/+
I:2
II:1
II:2
M/+
FBN1
M:c.4894C>T
c.4894C>T
II:2
TTTCCAGTGCTGCTGTCCAAC
I:1
TTTCCAGTGCCGCTGTCCAAC
F8
I:1
M/+
I:2
+/+
II:1
M/+
FBN1
M:c.7841C>T
c.7841C>T
II:1
TGCCTCAGCGYTCACATCTGC
I:1
TGCCTCAGCGYTCACATCTGC
I:2
TGCCTCAGCGCTCACATCTGC

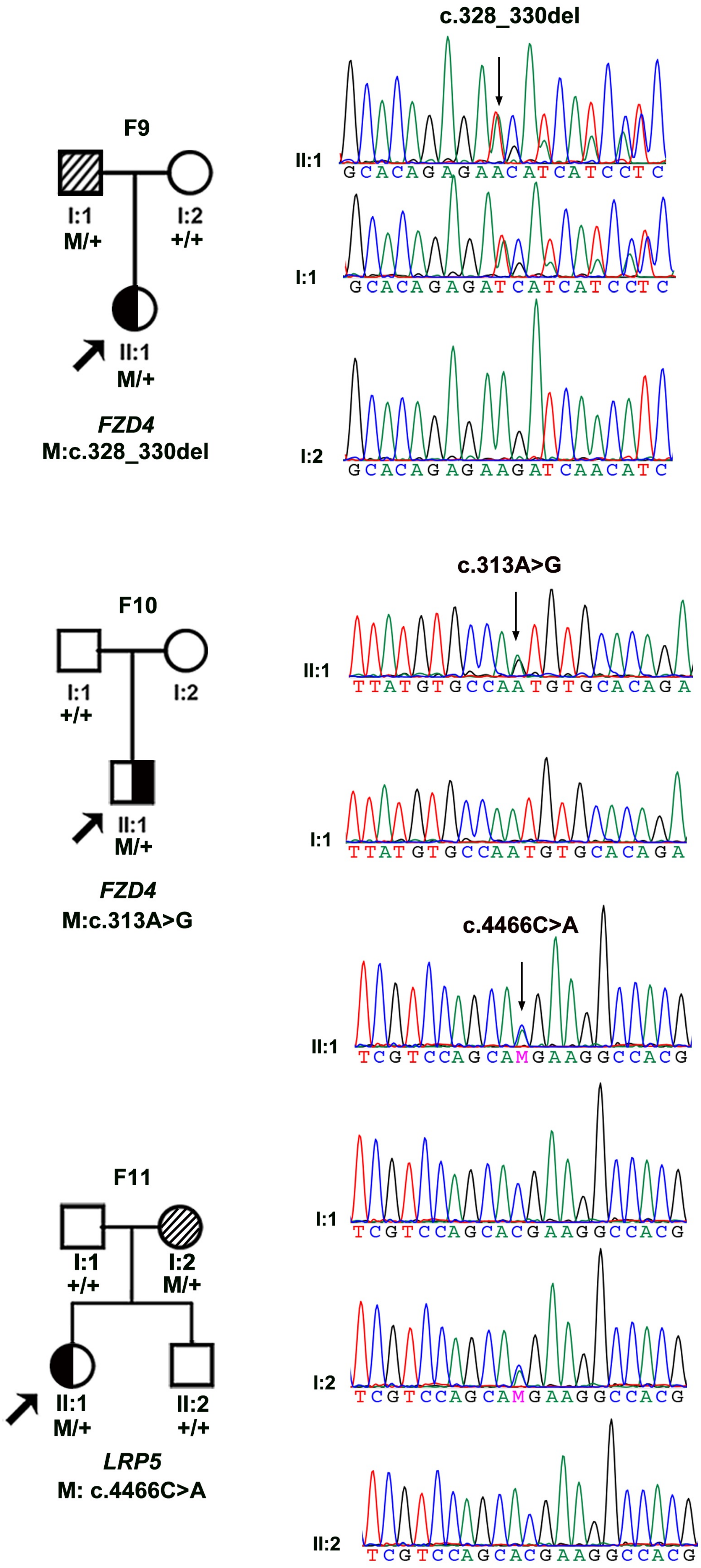
F9
I:1
M/+
I:2
+/+
II:1
M/+
FZD4
M:c.328_330del
c.328_330del
II:1
GCACAGAGAACATCATCCTC
I:1
GCACAGAGATCATCATCCTC
I:2
GCACAGAGAAGATCAACATC
F10
I:1
+/+
I:2
II:1
M/+
FZD4
M:c.313A>G
c.313A>G
II:1
TTATGTGCCAATGTGCACAGA
I:1
TTATGTGCCAATGTGCACAGA
F11
I:1
+/+
I:2
M/+
II:1
M/+
II:2
+/+
LRP5
M: c.4466C>A
c.4466C>A
II:1
TCGTCCAGCAMGAAGGCCACG
I:1
TCGTCCAGCACGAAGGCCACG
I:2
TCGTCCAGCAMGAAGGCCACG
II:2
TCGTCCAGCACGAAGGCCACG

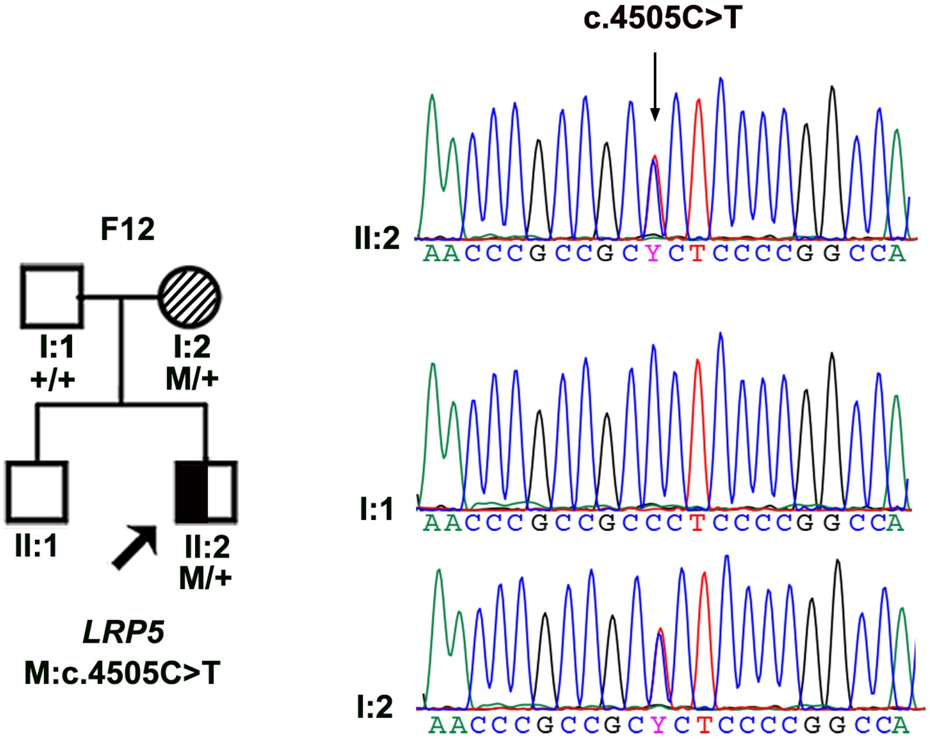
c.4505C>T
II:2
AACCCGCCGCYCTCCCCGGCCA
I:1
AACCCGCCGCCCTCCCCGGCCA
I:2
AACCCGCCGCYCTCCCCGGCCA
F12
I:1
+/+
I:2
M/+
II:1
II:2
M/+
LRP5
M:c.4505C>T

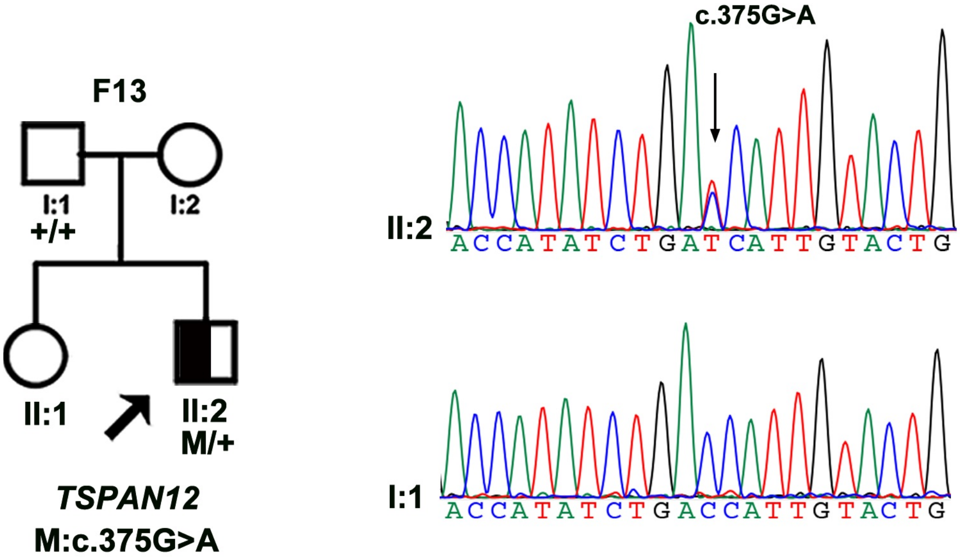
c.375G>A
II:2
ACCATATCTGATCATTGTACTG
I:1
ACCATATCTGACCATTGTACTG
F13
I:1
+/+
I:2
II:1
II:2
M/+
TSPAN12
M:c.375G>A

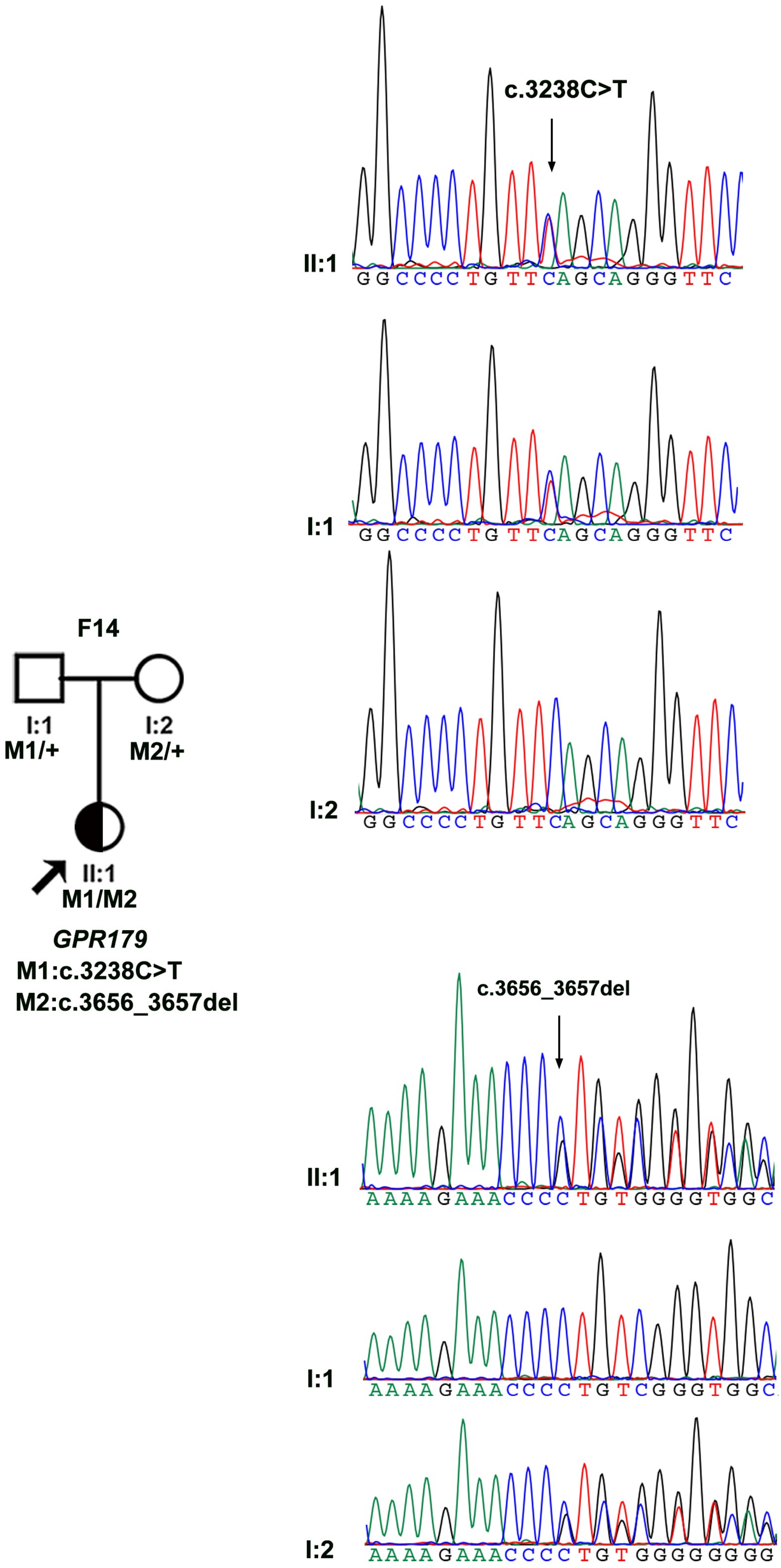
F14
I:1
M1/+
I:2
M2/+
II:1
M1/M2
GPR179
M1:c.3238C>T
M2:c.3656_3657del
c.3238C>T
II:1
GGCCCCTGTTCAGCAGGGTTC
I:1
GGCCCCTGTTCAGCAGGGTTC
I:2
GGCCCCTGTTCAGCAGGGTTC
c.3656_3657del
II:1
AAAAGAAACCCCTGTGGGGTGGC
I:1
AAAAGAAACCCCTGTCGGGTGGC
I:2
AAAAGAAACCCCTGTGGGGGGGG

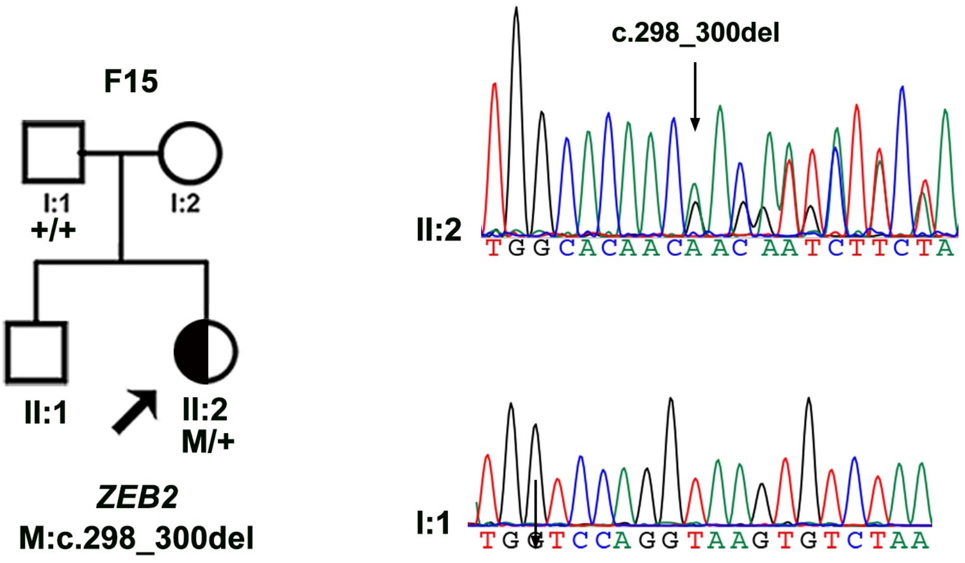
F15
I:1
+/+
I:2
II:1
II:2
M/+
ZEB2
M:c.298_300del
c.298_300del
II:2
I:1

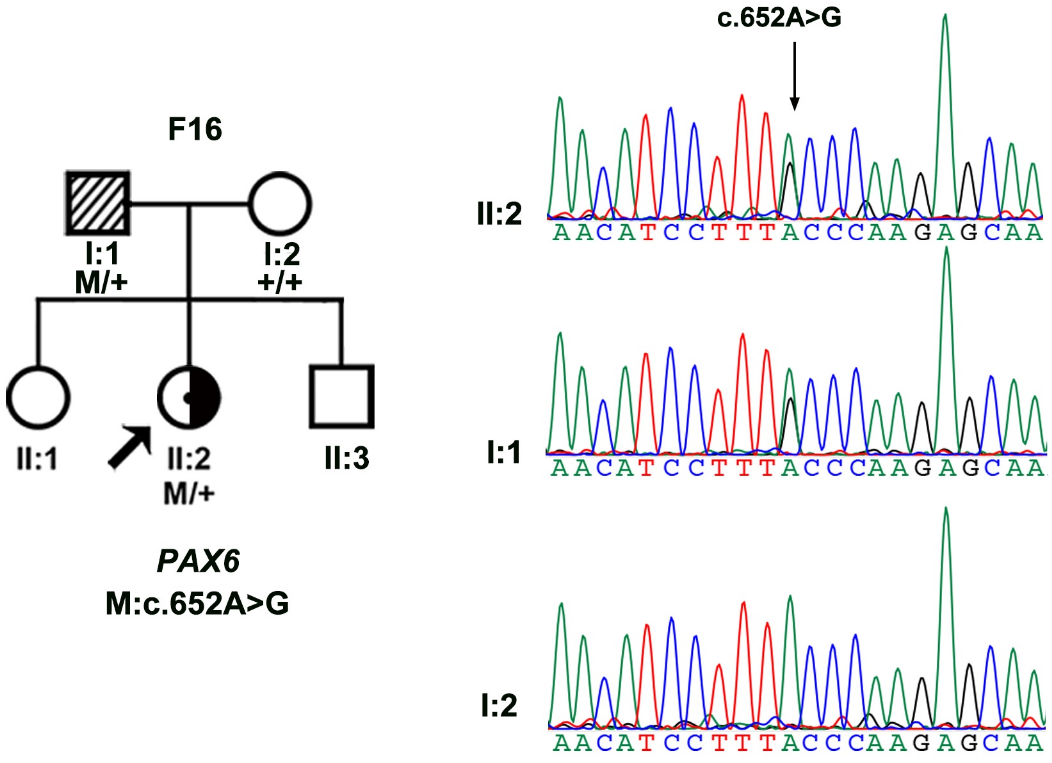
F16
I:1
M/+
I:2
+/+
II:1
II:2
M/+
II:3
PAX6
M:c.652A>G
c.652A>G
II:2
I:1
I:2

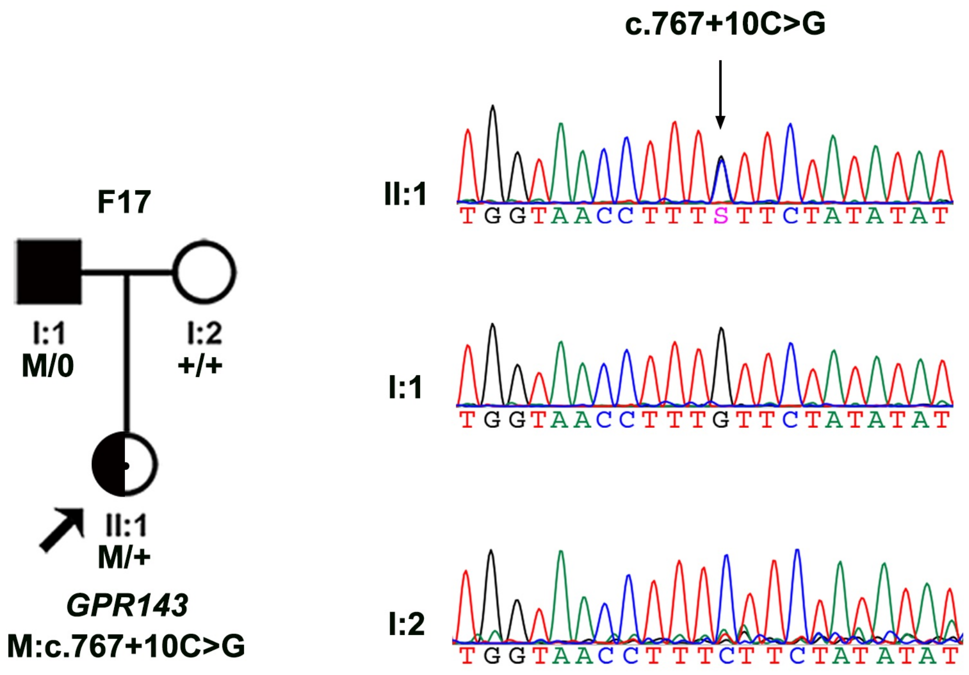

c.767+10C>G
F17
II:1
TGGTAACCTTTSTTCTATATAT
I:1
TGGTAACCTTTGTTCTATATAT
I:2
TGGTAACCTTTCTTCTATATAT
I:1
M/0
I:2
+/+
II:1
M/+
GPR143
M:c.767+10C>G

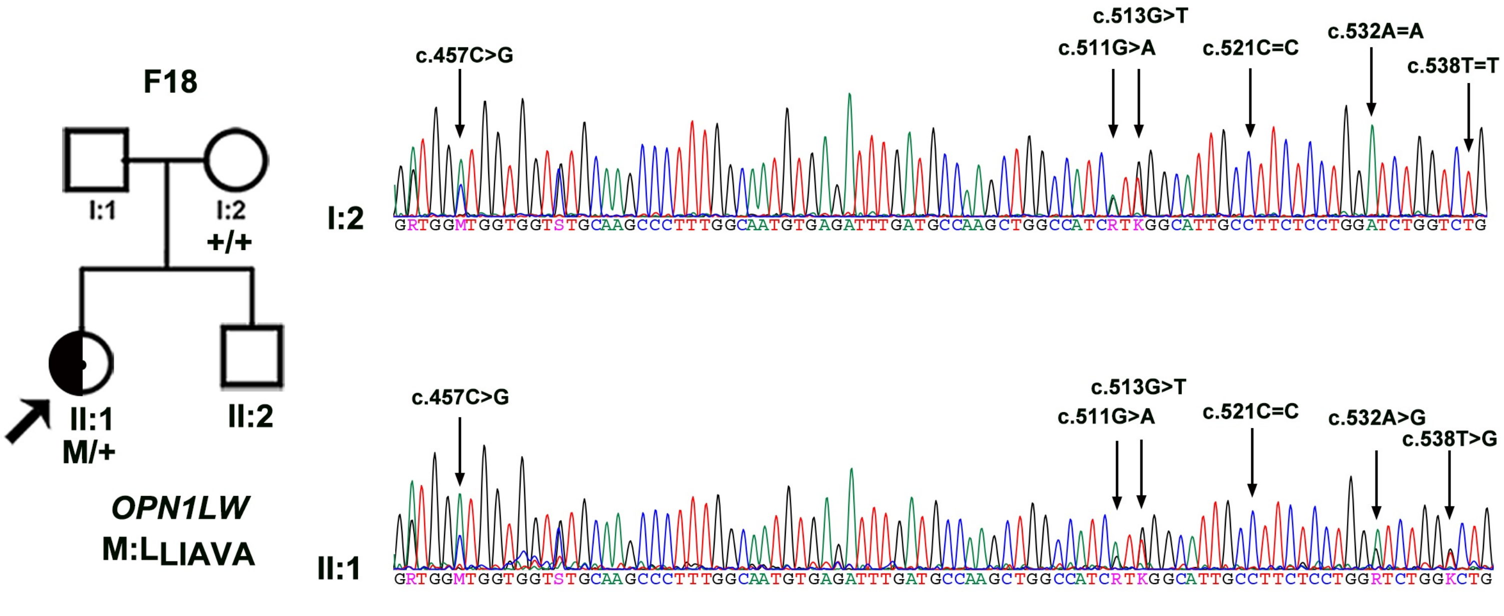

F18
I:1
I:2
+/+
II:1
M/+
II:2
OPN1LW
M:LLIAVA
c.457C>G
c.513G>T
c.511G>A
c.521C=C
c.532A=A
c.538T=T
GRTGGMTGGTGGTSTGCAAGCCCTTTGGCAATGTGAGATTTGATGCCAAGCTGGCCATCRTKGGCATTGCCTTCTCCTGGATCTGGTCTG
c.457C>G
c.513G>T
c.511G>A
c.521C=C
c.532A>G
c.538T>G
GRTGGMTGGTGGTSTGCAAGCCCTTTGGCAATGTGAGATTTGATGCCAAGCTGGCCATCRTKGGCATTGCCTTCTCCTGGRTCTGGKCTG

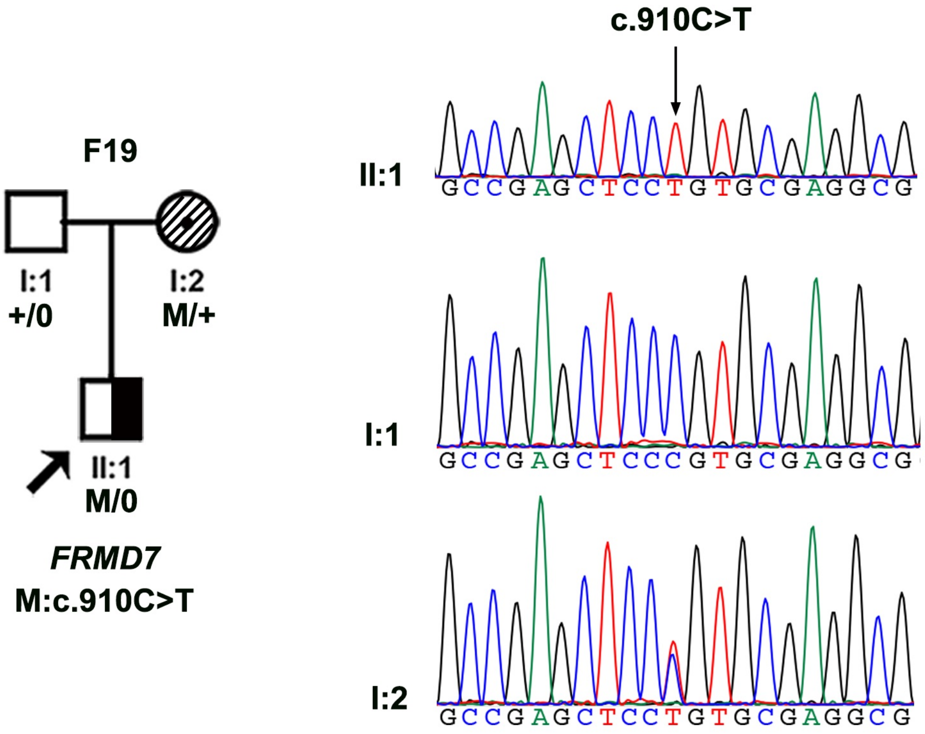
F19
I:1
+/0
I:2
M/+
II:1
M/0
FRMD7
M:c.910C>T
c.910C>T
II:1
GCCGAGCTCCTGTGCGAGGCG
I:1
GCCGAGCTCCCGTGCGAGGCG
I:2
GCCGAGCTCCTGTGCGAGGCG

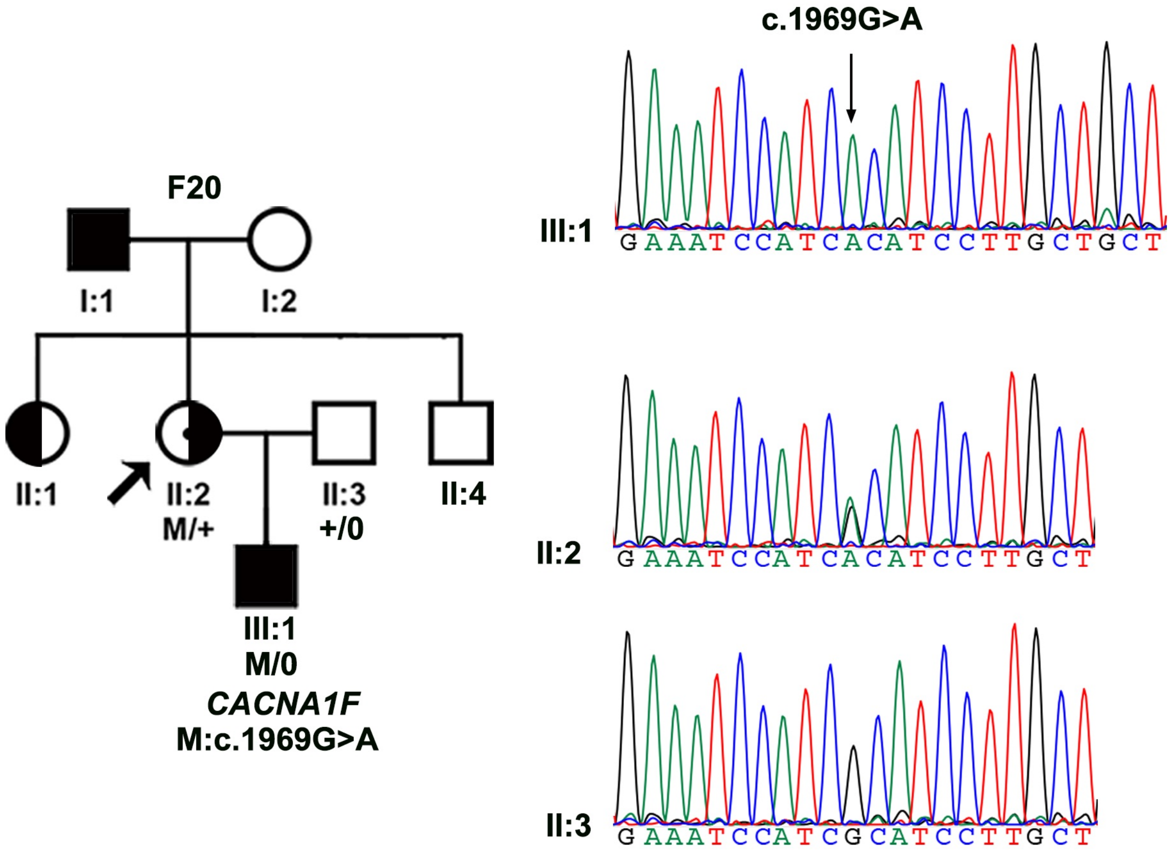
F20
I:1
I:2
II:1
II:2
M/+
II:3
+/0
II:4
III:1
M/0
CACNA1F
M:c.1969G>A
c.1969G>A
III:1
GAAATCCATCACATCCTTGCTGCT
II:2
GAAATCCATCACATCCTTGCT
II:3
GAAATCCATCGCATCCTTGCT
