# Supplementary material for: Clinical and genetic risk factors underlying severe consequence identified in 75 families with unilateral high myopia
Source: J Transl Med. 2024 Jan 19;22:75. doi: 10.1186/s12967-024-04886-5 (PMC10797748; doi:10.1186/s12967-024-04886-5)
Supplement: Supplementary file 3 — Additional file 3: Figure S3. Representative image of each peripheral retinal change identified in the study. Table S6. List of peripheral retinal changes in this cohort. [file 12967_2024_4886_MOESM3_ESM.pdf]

Figure S3. Representative image of each peripheral retinal change identified in the study.

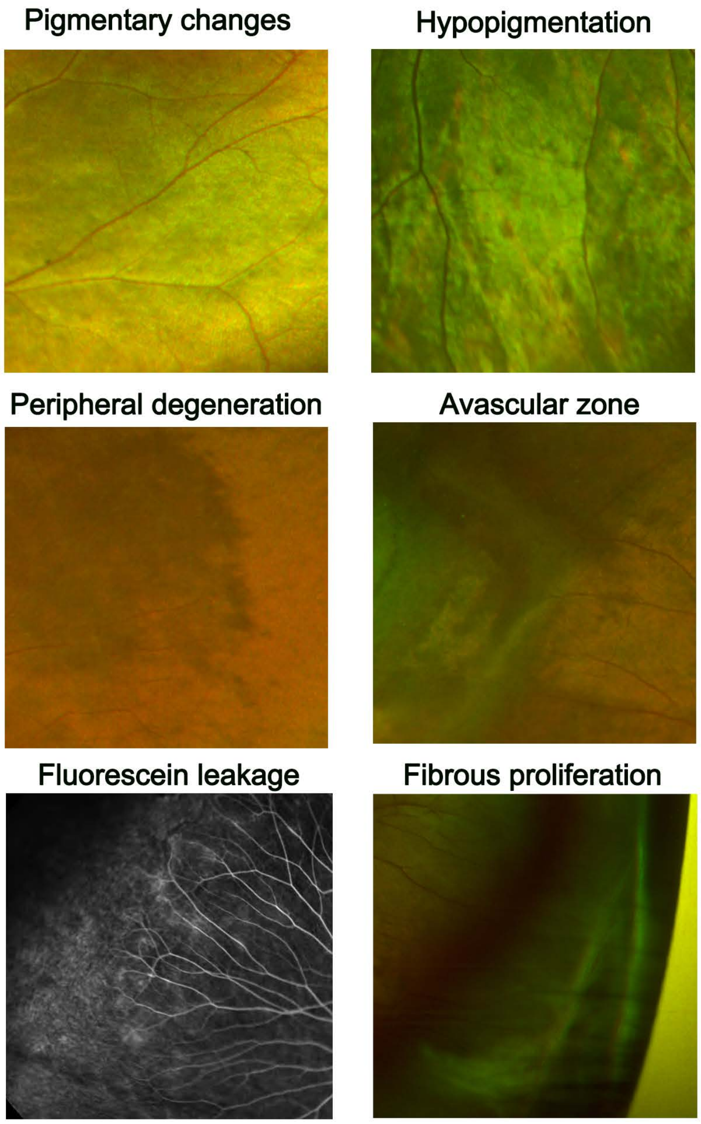

Table S6. List of peripheral retinal changes in this cohort

**Peripheral Retinal Abnormalities List**
Peripheral Pigmentary changes
Hypopigmentation
Lattice degeneration
Peripheral retinal degeneration
Peripheral avascular zone
Neovascularization
Fluorescein leakage (FFA)
Fibrous proliferation
